# Supplementary figures and images for: The Ync13–Rga7–Rng10 complex selectively coordinates secretory vesicle trafficking and secondary septum formation during cytokinesis
Source: PLoS Biol. 2025 Oct 27;23(10):e3003466. doi: 10.1371/journal.pbio.3003466 (PMC12574955; doi:10.1371/journal.pbio.3003466)

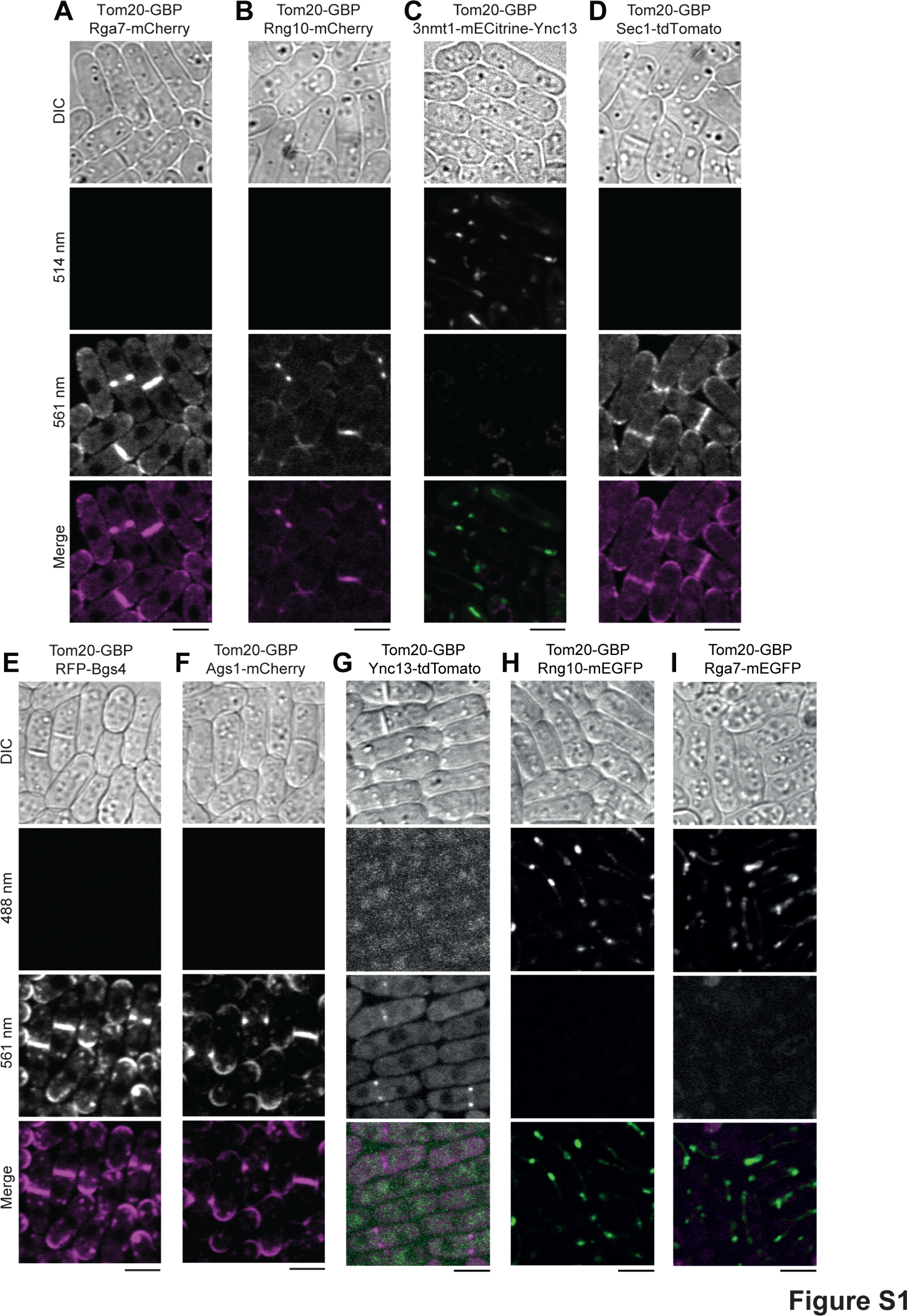

Supplement: S1 Fig — (A–I) Micrographs of DIC, 514/488/561 nm channels, and merged channels showing cells expressing Tom20-GBP and another indicated protein. Tom20-GBP does not bind to mCherry, RFP, or tdTomato so the tagged proteins cannot be recruited to mitochondria without proteins tagged with mEGFP or mECitrine. No signal bleed-through between red (561 nm)/yellow (514 nm) or red (561 nm)/green (488 nm) channels was detected. The brightness and contrast were adjusted the same as the experimental groups with three tagged proteins shown in Figs 1, S2, or S3 so some panels appear almost totally black. Bars, 5 μm. (TIF) [file pbio.3003466.s001.tif]

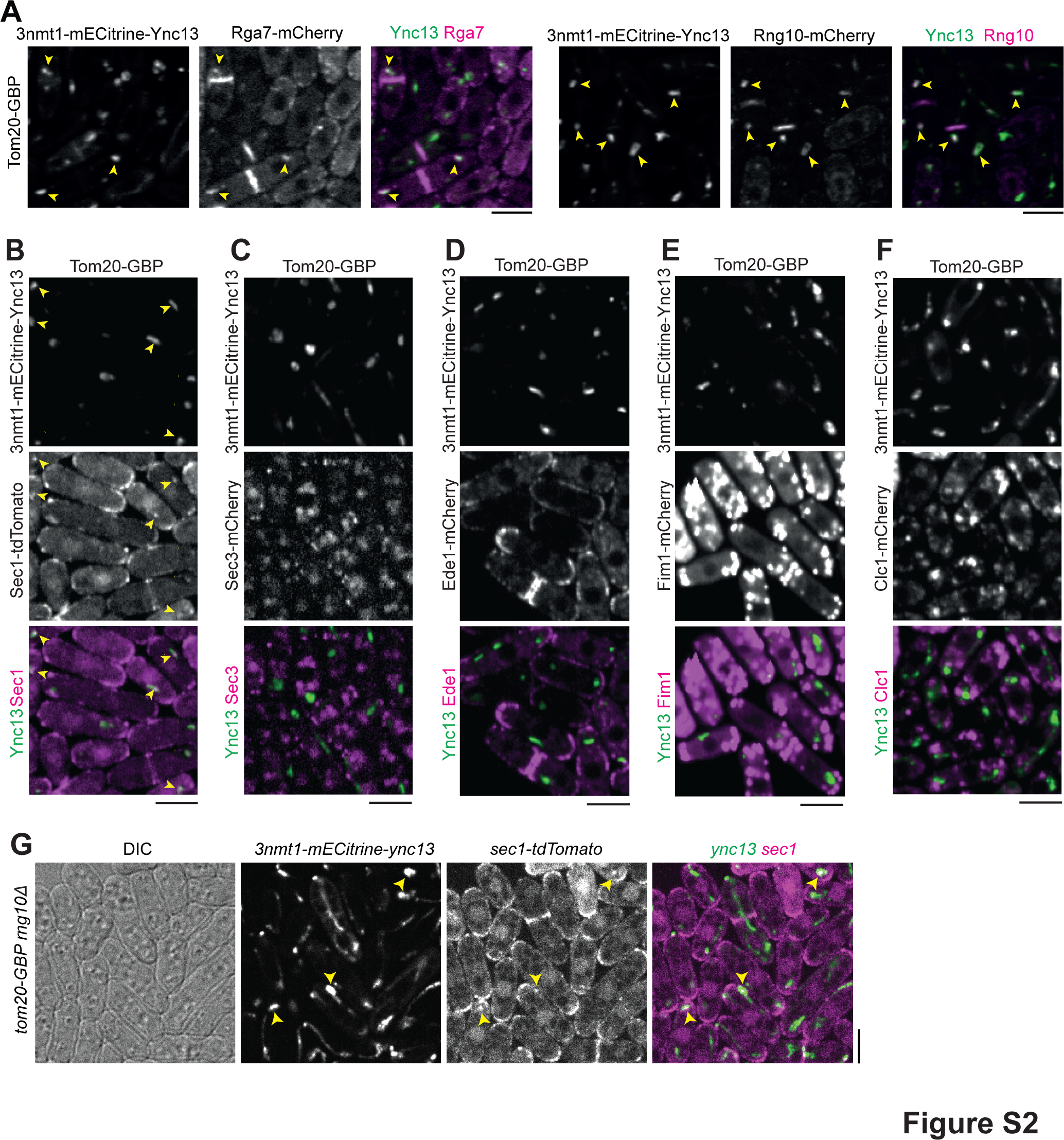

Supplement: S2 Fig — Mislocalized Ync13 ectopically targets (examples marked with arrowheads) Rga7 and Rng10 (A), and Sec1 (B) to mitochondria; but cannot interact with the exocyst subunit Sec3 (C) or endocytic proteins Ede1 (D), fimbrin Fim1 (E), or clathrin light chain Clc1 (F). Ync13 was mildly overexpressed using the 3nmt1 promoter by growing exponentially in YE5S liquid medium for ~48 h before imaging. (G) Mislocalized Ync13 ectopically targets Sec1 to mitochondria without Rng10 (examples marked with arrowheads). Cells were grown in YE5S + 1.2 M sorbitol + thiamine at 25°C for 24 h and then shifted to the same medium without thiamine to induce Ync13 expression for 16 h. Cells were washed twice with EMM5S and imaged on EMM5S gelatin pad. Bars, 5 μm. (TIF) [file pbio.3003466.s002.tif]

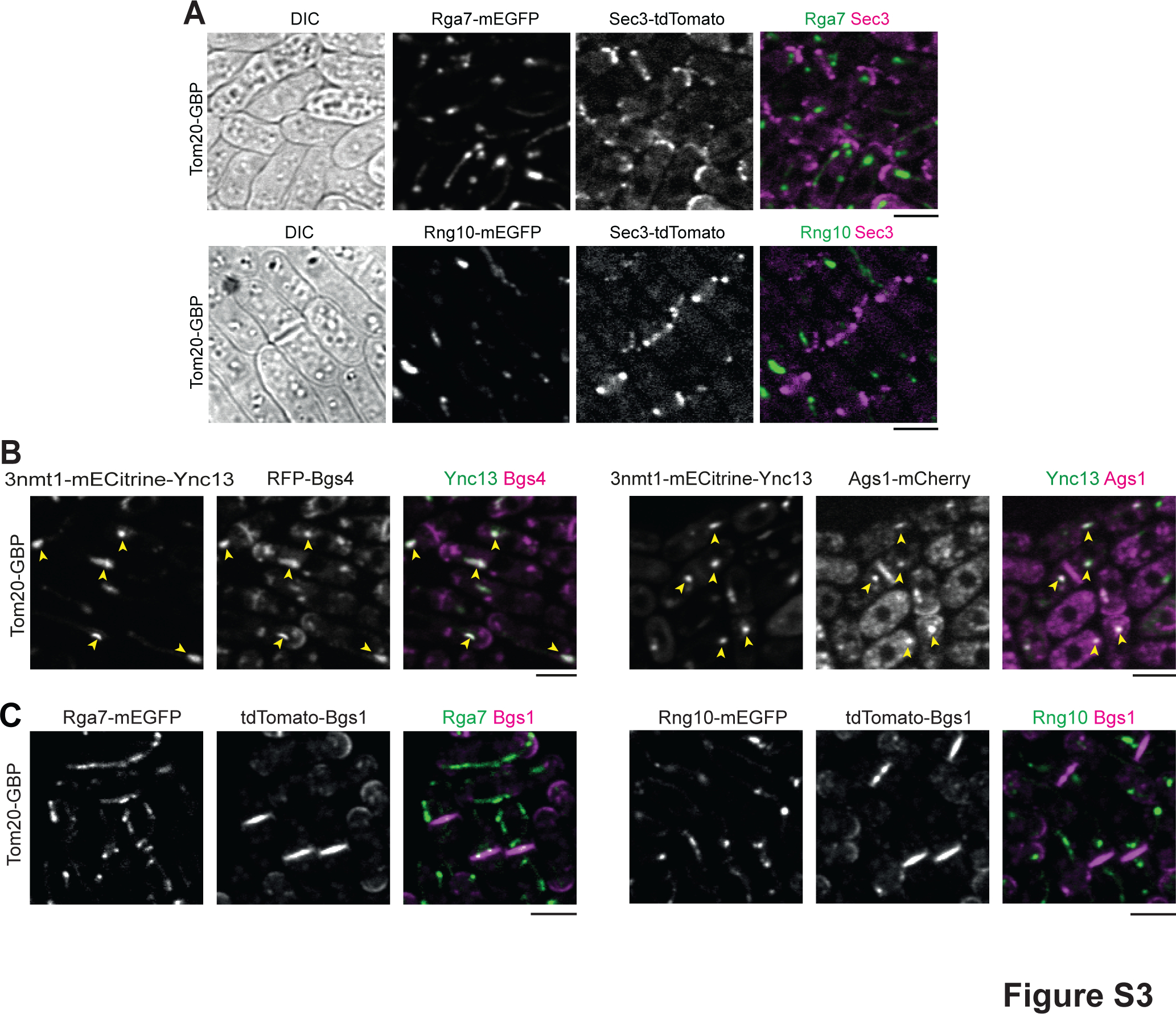

Supplement: S3 Fig — (A and C) Rga7 and Rng10 cannot mistarget the exocyst subunit Sec3 (A) or Bgs1 (C) to mitochondria. (B) Mislocalized Ync13 ectopically targets Bgs4 and Ags1 to mitochondria (examples marked with arrowheads). Ync13 was overexpressed using the 3nmt1 promoter by growing exponentially in YE5S liquid medium for ~48 h before imaging. Bars, 5 μm. (TIF) [file pbio.3003466.s003.tif]

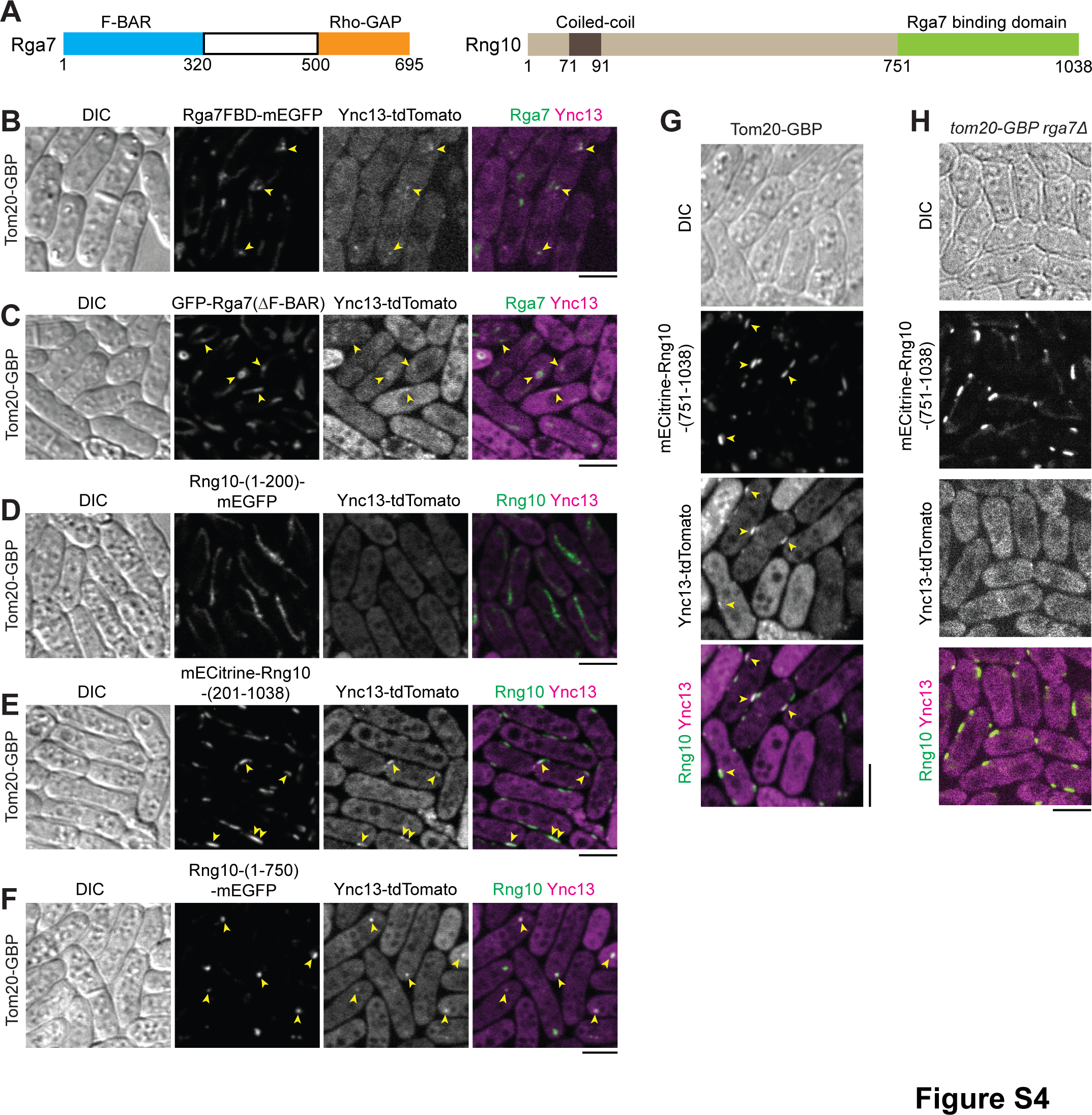

Supplement: S4 Fig — (A) Domain schematics of Rga7 and Rng10 [74,75]. (B–G) Arrowheads mark examples of colocalization at mitochondria. Except Rng10-(1-200) in (D), all other Rga7 and Rng10 truncations (B, C, and E–G) can mistarget Ync13-tdToamto to mitochondria. Rga7FBD = Rga7(1-320) [76]; Rga7(ΔF-BAR), Rga7 without the F-BAR domain. (H) mECitrine-Rng10(751-1038) cannot mistarget Ync13 to mitochondria without Rga7. Cells were grown in YE5S + 1.2 M sorbitol at 25°C for 36 h and then washed twice with EMM5S and imaged on EMM5S gelatin pad. Bars, 5 μm. (TIF) [file pbio.3003466.s004.tif]

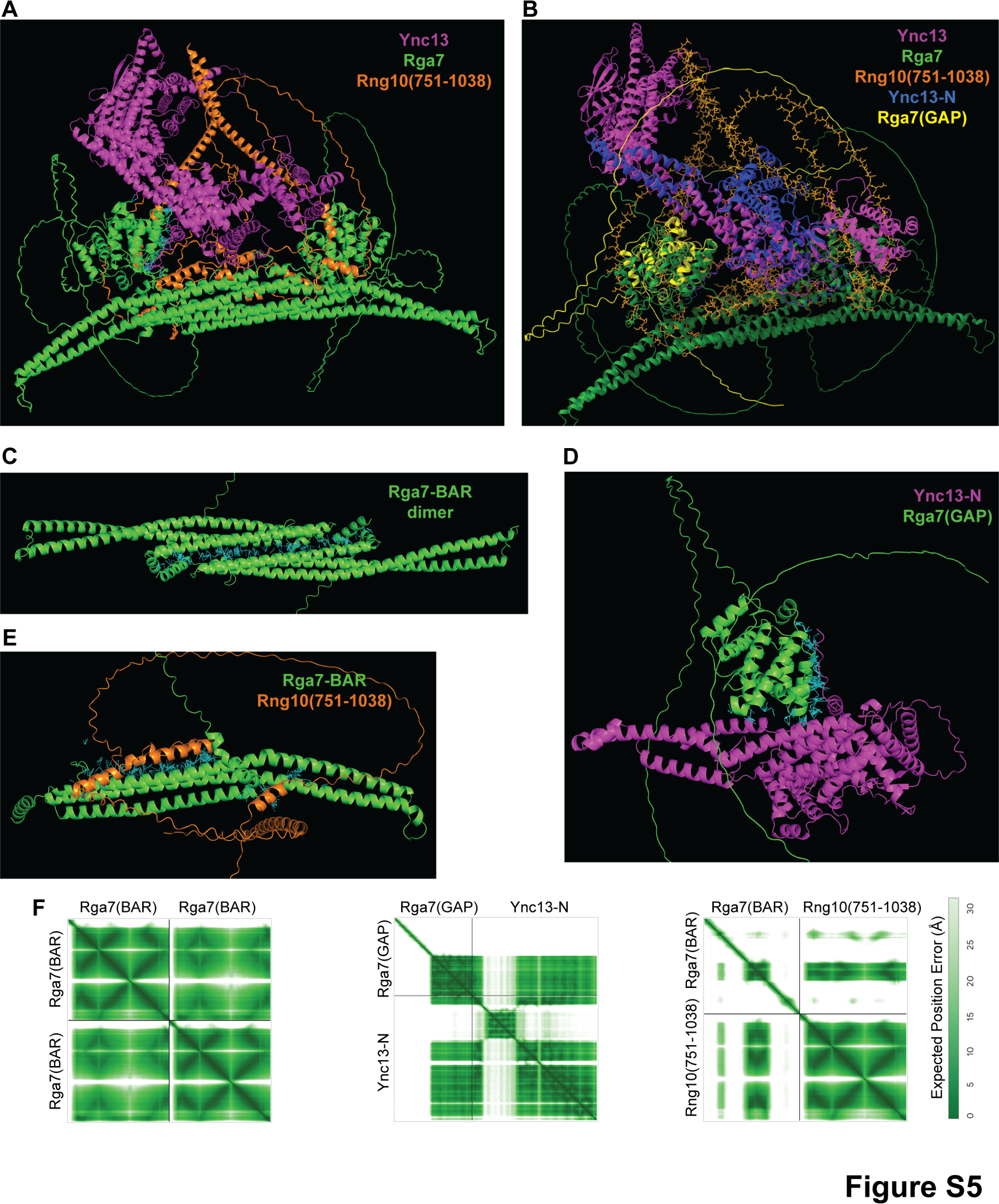

Supplement: S5 Fig — (A) Ribbon diagram of the predicted Rga7–Rng10–Ync13 complex, illustrating the overall domain organization and relative orientation of the three proteins. For the modeling, one copy of FL Ync13 (magenta), the dimer of FL Rga7 (green), and two copies of Rng10(aa751-1038) (orange) were used. The contacts between residues with a distance <4 Å are colored in cyan in (A–E). (B) Overlay (Root Mean Square Deviation: RMSD = 0.761 Å) of predicted structures of Ync13 N-terminus (aa 1-600) and Rga7-GAP (aa 321-695) domain panel (D) onto the whole complex in panel (A). Here, Ync13-N is colored in blue and Rga7(GAP) in yellow from (D) while all the original colors for the whole complex from (A). (C–E) Ribbon diagram of predicted interaction between (C) Rga7 F-BAR dimer (pTM: 0.74, ipTM: 0.71); (D) Ync13 N-terminal (1-600 aa) and Rga7-GAP domain (321-695 aa) (pTM: 0.63, ipTM: 0.64); and (E) Rga7 F-BAR and Rng10(751-1038) (pTM: 0.56, ipTM: 0.78). (F) The PAE (Predicted Aligned Error) plot for the AlphaFold3 predictions in (C–E). (TIF) [file pbio.3003466.s005.tif]

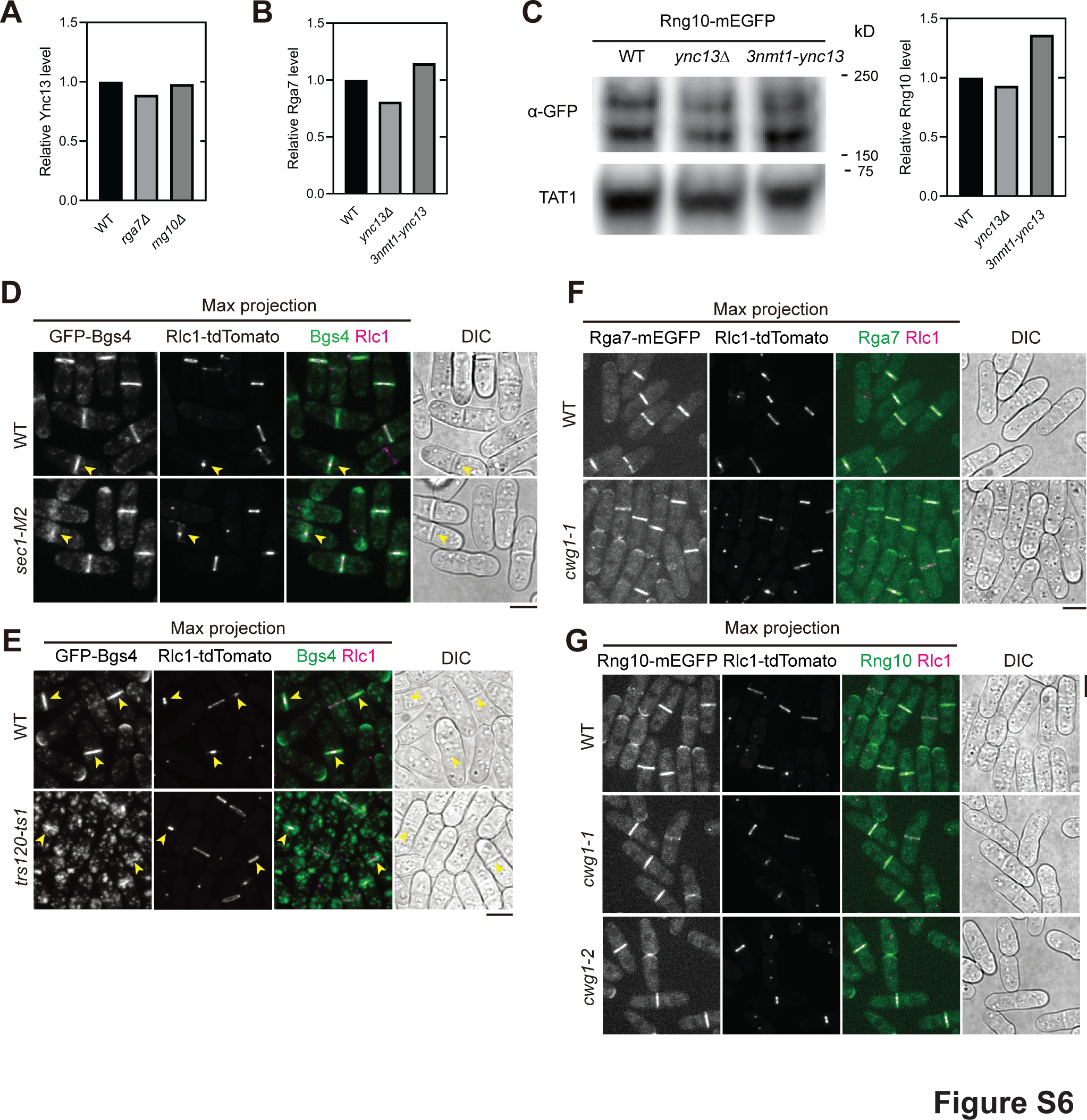

Supplement: S6 Fig — (A and B) Protein levels of Ync13 and Rga7 from the Western blots associated with Fig 3C (A) and Fig 3D (B). (C) Western blot and quantification of Rng10 levels in the indicated strains. (D–G) Cells grown exponentially at 25°C were shifted to 36°C for 4 h (D, F, G) or 2 h (E) before imaging. Rlc1-tdTomato as the ring marker. Bgs4 localization in sec1-M2 (D) or trs120-ts1 (E) mutant cells. Rga7 (F) and Rng10 (G) localization in bgs4 mutants cwg1-1 and cwg1-2. The underlying data (for panels A–C) can be found in S1 Raw Data file and uncropped Western blots (for panel C) in S1 Raw Images. Bars, 5 μm. (TIF) [file pbio.3003466.s006.tif]

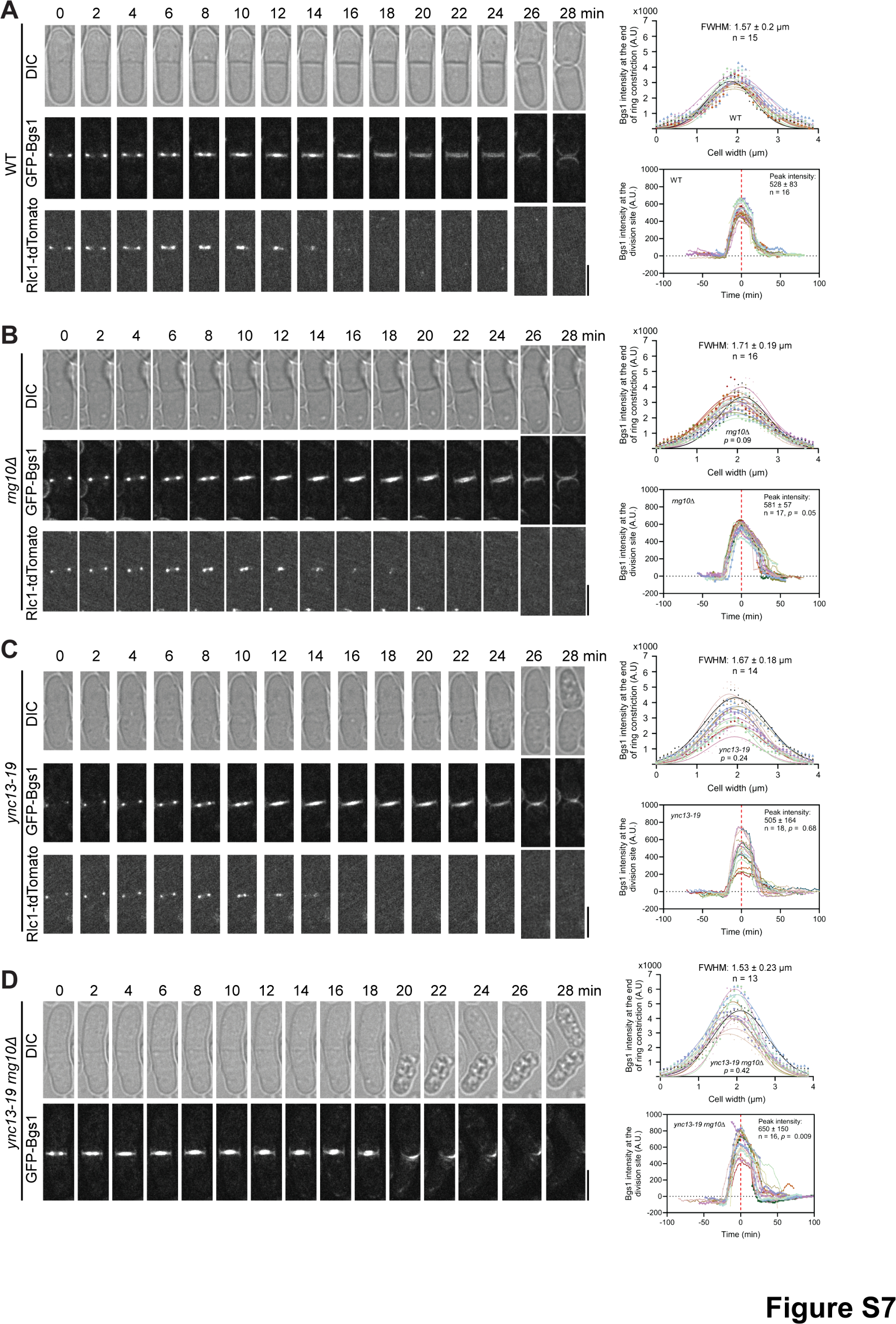

Supplement: S7 Fig — (A–D) Time course of DIC and middle focal plane of fluorescence images (left, in min), Bgs1 distribution (measured by FWHM) along the division site at the end of ring constriction (top right), and Bgs1 intensity at the division site over time (bottom right) in (A) WT, (B) rng10Δ, (C) ync13-19, and (D) rng10Δ ync13-19 cells. One (C) or both daughter cells (D) lysed after cell separation. Cells were grown exponentially in YE5S + 1.2 M sorbitol liquid media at 25°C for 36 h, then washed and grown in YE5S without sorbitol at 36°C for 2 h before imaging at 36°C in imaging dish covered with YE5S agar medium. Thirteen slices spaced at 0.5 μm at each time point were taken every 2 min for 2 h. Bgs1 intensity at the division site was measured using sum intensity projections and the background subtracted. Line scans along the division site were fitted in Gaussian distribution to calculate FWHM (mean ± SD) for cells at the end of ring constriction (the single time point when the ring has constricted to a dot in cell center and Rlc1 has reached the highest pixel intensity). Bgs1 intensity profiles at the division site over time were plotted and cells were aligned using the end of Rlc1 ring constriction (A–C) or peak Bgs1 intensity (D). P-values were from Welch’s t test. The underlying data (for panels A–D) can be found in S1 Raw Data file. Bars, 5 μm. (TIF) [file pbio.3003466.s007.tif]

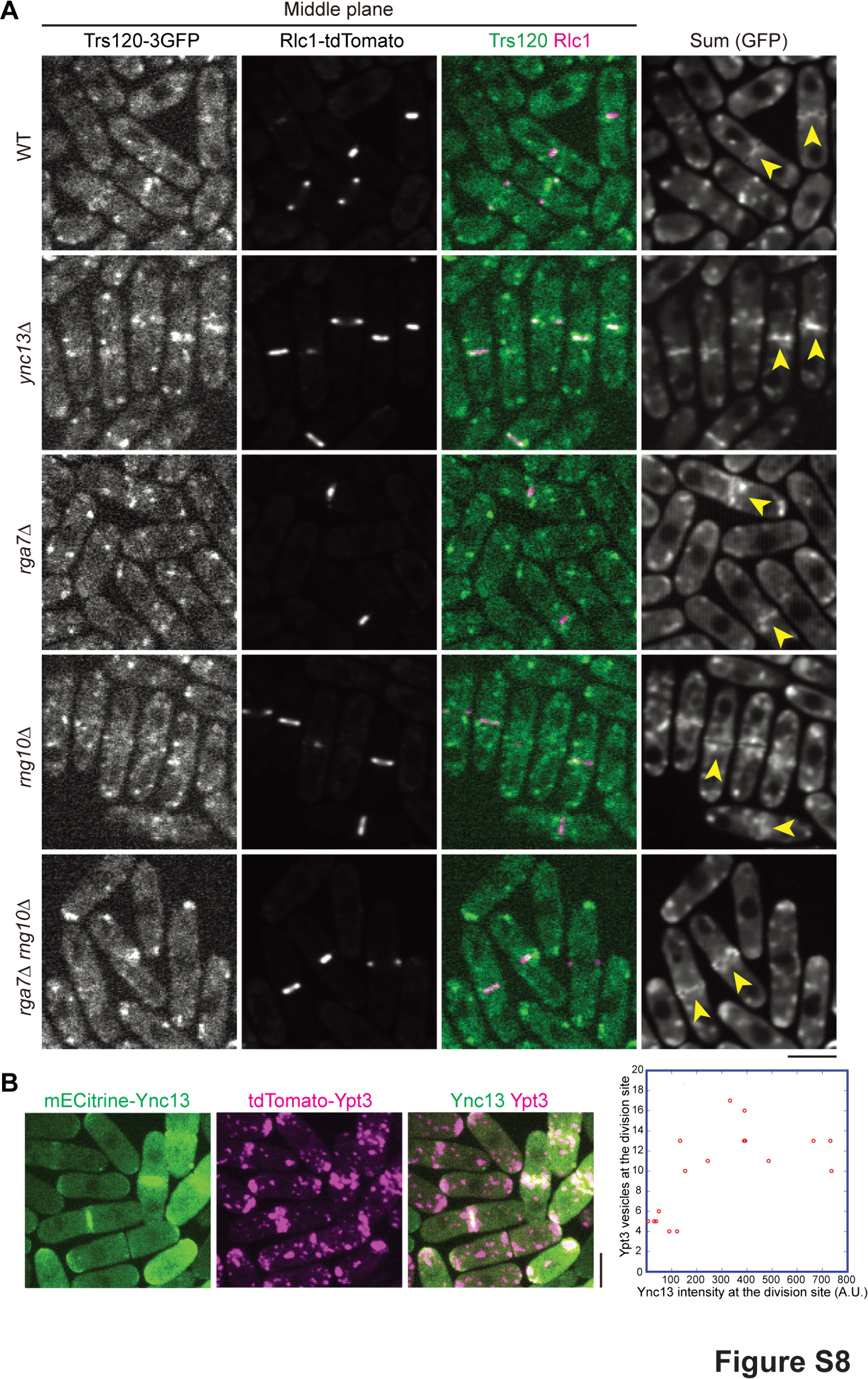

Supplement: S8 Fig — (A) Rlc1-tdTomato marks the position and diameter of the contractile ring. Trs120-3GFP accumulates inside the area with the ring in ync13Δ cells, but outside the ring area in rga7Δ, rng10Δ, and rga7Δ rng10Δ cells at the division site. The middle focal planes along with the sum intensity projection of the GFP channel from a 2-min continuous movie (exposure time 200 ms) without delay are shown. Arrowheads mark Trs120-3GFP in cells with constricting ring. Cells were grown exponentially in EMM5S liquid media for ~48 h before imaging. (B) Correlation between Ync13 intensity and the number of Ypt3 vesicles at the division site. (Left) Maximal intensity projection of cells expressing both 3nmt1-mECitrine-ync13 and tdTomato-ypt3. (Right) The number of Ypt3 vesicles arriving at the division site during the 2 min continuous movie vs. Ync13 intensity. Cells were grown in YE5S + thiamine liquid medium for 24 h and then grown in YE5S without thiamine for 16 h before imaging. The underlying data (for panel B) can be found in S1 Raw Data file. Bars, 5 μm. (TIF) [file pbio.3003466.s008.tif]

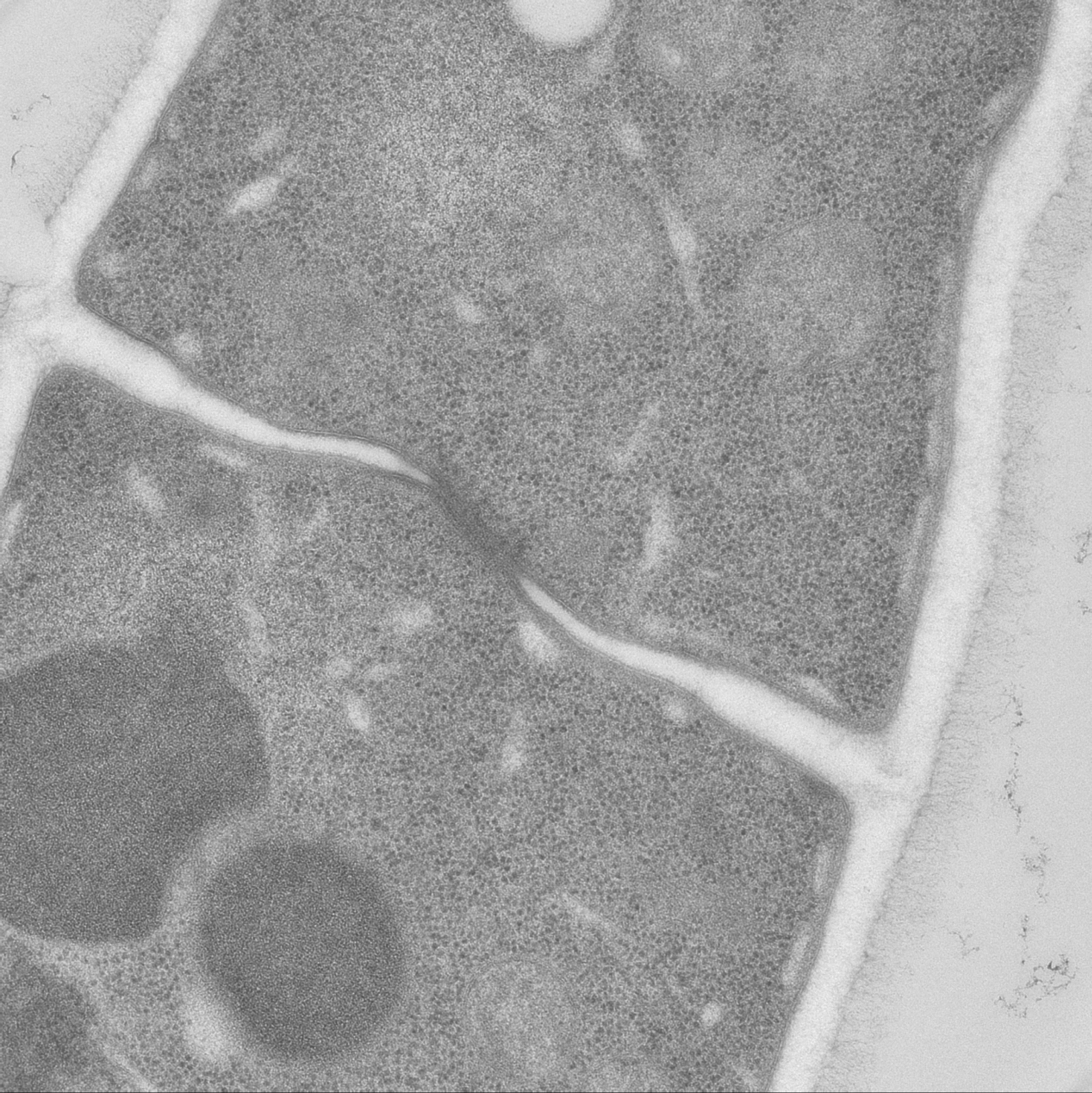

500 nm

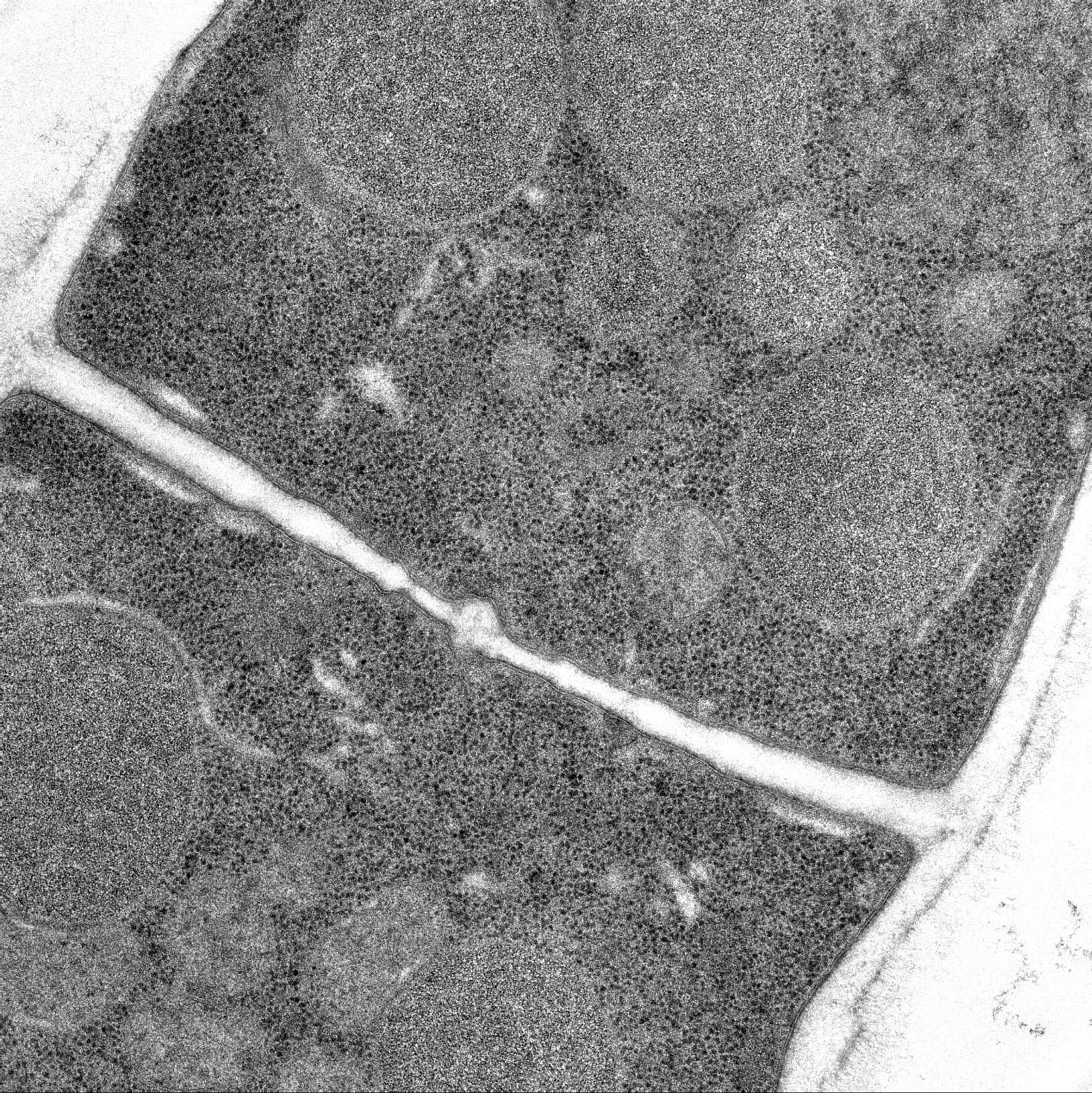

500 nm

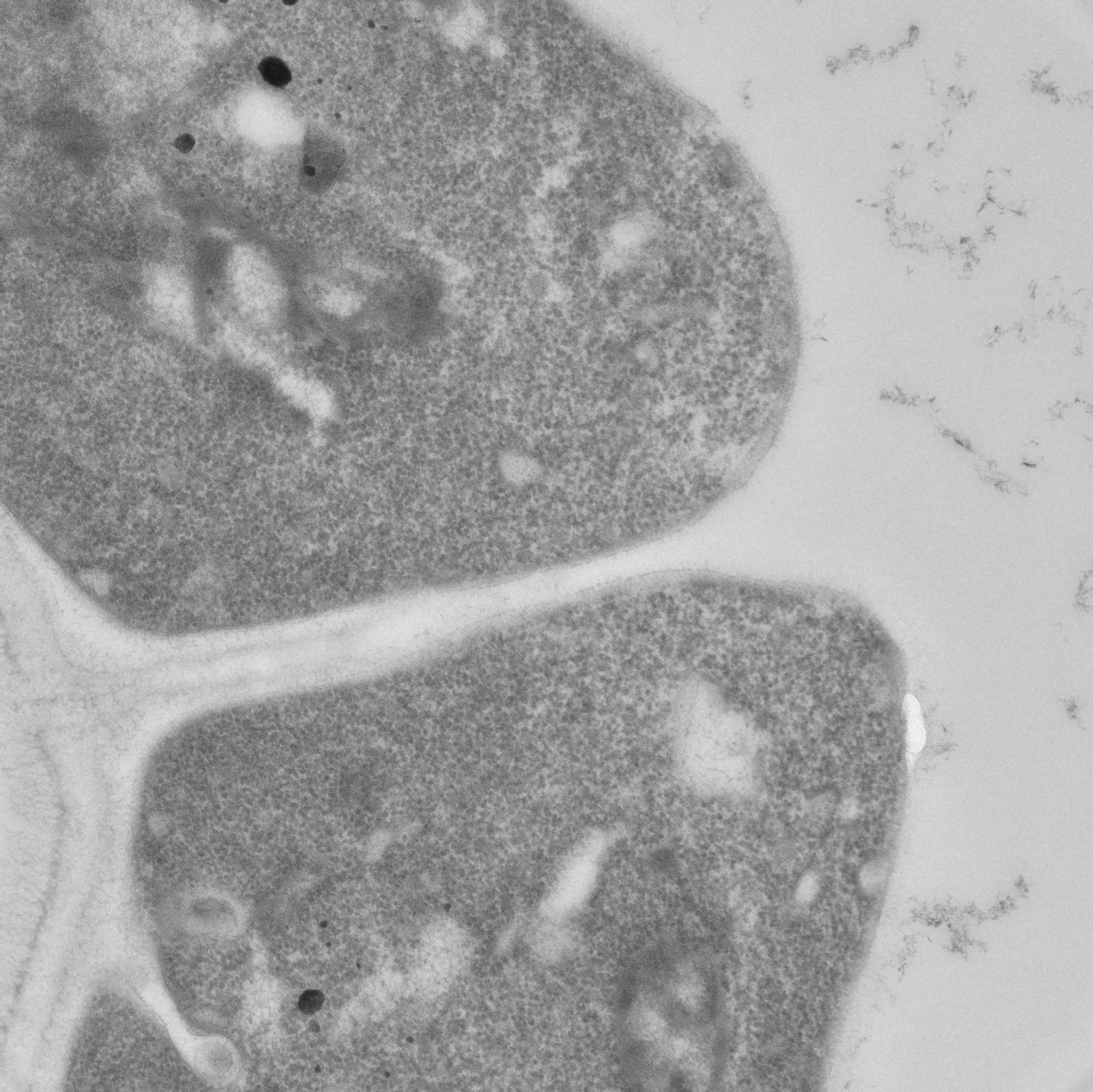

500 nm

Supplement: S2 Raw images — (PDF) [file pbio.3003466.s015.pdf]
