# Supplementary material for: The Ync13–Rga7–Rng10 complex selectively coordinates secretory vesicle trafficking and secondary septum formation during cytokinesis
Source: PLoS Biol. 2025 Oct 27;23(10):e3003466. doi: 10.1371/journal.pbio.3003466 (PMC12574955; doi:10.1371/journal.pbio.3003466)
Supplement: S1 Table — (DOCX) [file pbio.3003466.s012.docx]

**Supporting information**

**S1 Table. Proteins identified in mass spectrometry of affinity-purified Ync13-3xFlag from *S. pombe*^a^**

| Identified proteins | Exclusive unique  peptide count | Sequence coverage (%) |
| --- | --- | --- |
| Sec71 | 26 | 16% |
| Vps13b | 13 | 6% |
| Coy1 | 12 | 22% |
| Cit1 | 12 | 28% |
| Mdn1 | 11 | 3% |
| Bag102 | 10 | 56% |
| Ysp2 | 10 | 23% |
| Ggt1 | 9 | 18% |
| Dug1 | 8 | 26% |
| Mak3 | 8 | 5% |
| Elp3 | 8 | 14% |
| **Rng10** | **7** | **10%** |
| Los1 | 7 | 10% |
| SPBC16G5.07c | 7 | 25% |
| SPAP8A3.03 | 6 | 16% |
| Pmd1 | 6 | 6% |
| Myo52 | 6 | 6% |
| Fet4 | 6 | 17% |
| Oxa102 | 6 | 18% |
| Vtc2 | 6 | 11% |
| Bch1 | 6 | 15% |
| Otg3 | 6 | 25% |
| Gtb1 | 6 | 14% |
| Kap123 | 6 | 10% |
| Cap1 | 5 | 17% |
| SPBC713.03 | 5 | 13% |
| Grx4 | 5 | 40% |
| Eca39 | 5 | 19% |
| Etp1 | 5 | 11% |
| Lap2 | 5 | 10% |
| Rnc1 | 5 | 20% |
| Yta4 | 5 | 16% |
| Erg25 | 5 | 16% |
| **Rga7** | **5** | **8%** |
| Sec11 | 5 | 43% |
| Msh2 | 4 | 5% |
| Sal3 | 4 | 6% |

^a^Protein samples purified from the strain TP150 transformed with plasmids pREP3X-Rng13 FL-3Flag (JQW1041) or pREP3X-GGSGGS-3Flag (JQW1072) were analyzed by mass spectrometry. The proteins listed in this table were specifically identified in cells with the plasmid JQW1041.
