# Supplementary material for: The Ync13–Rga7–Rng10 complex selectively coordinates secretory vesicle trafficking and secondary septum formation during cytokinesis
Source: PLoS Biol. 2025 Oct 27;23(10):e3003466. doi: 10.1371/journal.pbio.3003466 (PMC12574955; doi:10.1371/journal.pbio.3003466)
Supplement: S2 Table — (DOCX) [file pbio.3003466.s013.docx]

**Supporting information**

**S2 Table. *S. pombe* strains used in this study.**

| **Strain** | **Genotype** | **Figure/video/**  **reference** |
| --- | --- | --- |
| JW6068 | *ync13-mECitrine-kanMX6 rga7-mCFP-kanMX6 ade6-210 leu1-32 ura4-D18* | Fig 1A |
| JW6128 | *rng10-mCherry-natMX6 ync13-mECitrine-kanMX6 ade6-210 leu1-32 ura4-D18* | Fig 1A |
| JW9010 | *rga7-mEGFP-kanMX6 ync13-tdTomato-natMX6 tom20-GBP-hphMX6 ade6-210 leu1-32 ura4-D18* | Fig 1B |
| JW8946 | *rng10-mEGFP-kanMX6 ync13-tdTomato-natMX6 tom20-GBP-hphMX6 ade6-M210 leu1-32 ura4-D18* | Fig 1B |
| JW9435 | *rga7-mEGFP-kanMX6 trs120-tdTomato-natMX6 tom20-GBP-hphMX6*  *ade6-21X leu1-32 ura4-D18* | Fig 1C |
| JW9339 | *rng10-mEGFP-kanMX6 trs120-tdTomato-natMX6 tom20-GBP-hphMX6*  *ade6-21X leu1-32 ura4-D18* | Fig 1C |
| JW9315 | *rga7-mEGFP-kanMX6 tom20-GBP-hphMX6 bgs4∆::ura4^+^*  *Pbgs4^+^::RFP-bgs4^+^-leu1^+^ ade6-M210 leu1-32 ura4-D18 his3-D1?* | Fig 1D |
| JW9345 | *rng10-mEGFP-kanMX6 tom20-GBP-hphMX6 bgs4∆::ura4^+^*  *Pbgs4^+^::RFP-bgs4^+^-leu1^+^ leu1-32 ura4-D18 his3-D1? ade6-M210?* | Fig 1D |
| JW9397 | *ags1∆ 3’UTR_ags1_^+^::ags1^+^-Cherry:leu1^+^:ura4^+^ rga7-mEGFP-kanMX6 tom20-GBP-hphMX6 ade6-M210 his3-D1? leu1-32 ura4-D18* | Fig 1E |
| JW9394 | *ags1∆ 3’UTR_ags1_^+^::ags1^+^-Cherry:leu1^+^:ura4^+^ rng10-mEGFP-kanMX6 tom20-GBP-hphMX6 ade6-M210 his3-D1? leu1-32 ura4-D18* | Fig 1E |
| JW10114 | *tom20-GBP-hphMX6 rga7-mEGFP-kanMX6 smi1-tdTomato-kanMX6 ade6-210 leu1-32 ura4-D18* | Fig 1F |
| JW7782 | *ync13-mECitrine-kanMX6 rga7-13Myc-hphMX6 ade6-210 leu1-32 ura4-D18* | Fig 2A |
| JW5730 | *h^+^ ync13-mECitrine-kanMX6 ade6-210 leu1-32 ura4-D18* | Fig 2A |
| JW6734 | *h^-^ rga7-13Myc-hphMX6 ade6-210 leu1-32 ura4-D18* | Fig 2A |
| JW9614 | *rga7-13Myc-hphMX6 bgs4∆::ura4^+^ Pbgs4^+^::GFP-bgs4^+^-leu1^+^ ade6-210? leu1-32 ura4-D18 his3-D1?* | Fig 2B |
| 562 | *h^+^ bgs4∆::ura4^+^ Pbgs4^+^::GFP-bgs4^+^-leu1^+^ leu1-32 ura4-D18 his3-D1* | Fig 2B; Cortes et al., 2005 |
| JW6734 | *h^-^ rga7-13Myc-hphMX6 ade6-210 leu1-32 ura4-D18* | Fig 2B |
| JW10167 | *h^+^ rga7-13Myc-hphMX6 smi1-mEGFP-kanMX6 ade6-M210 leu1-32 ura4-D18* | Fig 2C |
| JW8905 | *h^-^ smi1-mEGFP-kanMX6 ade6-210 leu1-32 ura4-D18* | Fig 2C |
| JW6112 | *h^+^ rga7-13Myc-hphMX6 ade6-M210 leu1-32 ura4-D18* | Fig 2C |
| JW10194 | *rga7-mEGFP-kanMX6 smi1-13Myc-hphMX6 ade6-M210 leu1-32 ura4-D18* | Fig 2C |
| JW10185 | *h^+^ smi1-13Myc-hphMX6 ade6-M210 leu1-32 ura4-D18* | Fig 2C |
| JW3660 | *h^-^ rga7-mEGFP-kanMX6 ade6-210 leu1-32 ura4-D18* | Fig 2C |
| JW5730 | *h^+^ ync13-mECitrine-kanMX6 ade6-210 leu1-32 ura4-D18* | Fig 3, A and B |
| JW8876 | *rga7∆::ura4^+^ ync13-mECitrine-kanMX6 ade6-M21X leu1-32 ura4-D18* | Fig 3, A and B |
| JW8912 | *rng10Δ::hphMX6 ync13-mECitrine-kanMX6 ade6-210 leu1-32 ura4-D18* | Fig 3, A and B |
| JW5969 | *ync13-mECitrine-kanMX6 rlc1-mCherry-natMX6 ade6-210 leu1-32 ura4-D18* | Fig 3C |
| JW8895 | *ync13-mECitrine-kanMX6 rlc1-mCherry-natMX6 rga7Δ::natMX6 ade6-M210 leu1-32 ura4-D18* | Fig 3C |
| JW8913 | *ync13-mECitrine-kanMX6 rlc1-mCherry-natMX6 rng10Δ::hphMX6 ade6-210 leu1-32 ura4-D18* | Fig 3C |
| JW3660 | *h^-^ rga7-mEGFP-kanMX6 ade6 leu1-32 ura4-D18* | Fig 3D |
| JW6063 | *h^-^ ync13Δ::kanMX6 rlc1-tdTomato-natMX6 rga7-mEGFP-kanMX6 ade6 leu1-32 ura4-D18* | Fig 3D |
| JW9470 | *kanMX6-3nmt1-ync13 rlc1-tdTomato-natMX6 rga7-mEGFP-kanMX6 ade6-M210 leu1-32 ura4-D18* | Fig 3D |
| JW3693 | *rga7-mEGFP-kanMX6 rlc1-tdTomato-natMX6 ade6-M210 leu1-32 ura4-D18* | Fig 3, E and F |
| JW6063 | *h^-^ rga7-mEGFP-kanMX6 rlc1-tdTomato-natMX6 ync13Δ::kanMX6 ade6 leu1-32 ura4-D18* | Fig 3, E and F |
| JW5899 | *rng10-mEGFP-kanMX6 rlc1-tdTomato-natMX6 ade6-M210 leu1-32 ura4-D18* | Fig 3G |
| JW9483 | *rng10-mEGFP-kanMX6 rlc1-tdTomato-natMX6 ync13Δ::kanMX6 ade6 leu1-32 ura4-D18* | Fig 3G |
| JW6810 | *rlc1-tdTomato-natMX6 ags1∆ 3’UTR_ags1_^+^::ags1^+^-GFP:leu1^+^:ura4^+^ade6 leu1-32 ura4-D18* | Fig 4A |
| JW6808 | *ync13Δ::kanMX6 rlc1-tdTomato-natMX6 ags1∆ 3’UTR_ags1_^+^::ags1^+^-GFP:leu1^+^:ura4^+^ade6 leu1-32 ura4-D18* | Fig 4A |
| JW6152 | *bgs4∆::ura4^+^ Pbgs4^+^::GFP-bgs4^+^-leu1^+^ rlc1-tdTomato-natMX6 ync13Δ::kanMX6 leu1-32 ura4-D18  his3-D1? ade6?* | Fig 4, B and D |
| JW6153 | *bgs4∆::ura4^+^ Pbgs4^+^::GFP-bgs4^+^-leu1^+^ rlc1-tdTomato-natMX6 leu1-32 ura4-D18  his3-D1? ade6?* | Fig 4, B and D |
| JW5249 | *GFP-bgs1-leu1^+^ bgs1Δ::ura4^+^ rlc1-tdTomato-natMX6 ade6-M210 leu1-32 ura4-D18* | Fig 4, C and E |
| JW6616 | *GFP-bgs1-leu1^+^ bgs1Δ::ura4^+^ rlc1-tdTomato-natMX6 ync13Δ::kanMX6 ade6 ura4-D18* | Fig 4, C and E |
| 562 | *h^+^ bgs4∆::ura4^+^ Pbgs4^+^::GFP-bgs4^+^-leu1^+^ leu1-32 ura4-D18 his3-D1* | Fig 4F; Cortes et al., 2005 |
| JW6752 | *rng10Δ::kanMX6 bgs4∆::ura4^+^ Pbgs4^+^::GFP-bgs4^+^-leu1^+^ ura4-D18 his3-D1* | Fig 4F |
| JW9061 | *ync13-19-his5^+^-kanMX6 rng10Δ::kanMX6 bgs4∆::ura4^+^ Pbgs4^+^::GFP-bgs4^+^-leu1^+^ ade6? ura4 his3-D1? leu1-32?* | Fig 4F |
| JW9062 | *ync13-19-his5^+^-kanMX6 bgs4∆::ura4^+^ Pbgs4^+^::GFP-bgs4^+^-leu1^+^ ade6? ura4 his3-D1? leu1-32?* | Fig 4F |
| JW6152 | *ync13Δ::kanMX6 bgs4∆::ura4^+^ Pbgs4^+^::GFP-bgs4^+^-leu1^+^ rlc1-tdTomato-natMX6 leu1-32 ura4-D18  his3-D1? ade6?* | Fig 4G |
| JW6153 | *bgs4∆::ura4^+^ Pbgs4^+^::GFP-bgs4^+^-leu1^+^ rlc1-tdTomato-natMX6 leu1-32 ura4-D18  his3-D1? ade6?* | Fig 4G |
| JW9428 | *rng10Δ::hphMX6 rga7Δ::kanMX6 bgs4∆::ura4^+^ Pbgs4^+^::GFP-bgs4^+^-leu1^+^ rlc1-tdTomato-natMX6 ura4-D18 leu1-32 his3-D1? ade6?* | Fig 4G |
| JW9432 | *rng10Δ::hphMX6 bgs4∆::ura4^+^ Pbgs4^+^::GFP-bgs4^+^-leu1^+^ rlc1-tdTomato-natMX6 ura4? leu1-32 his3-D1? ade6?* | Fig 4G |
| JW9430 | *rga7Δ::kanMX6 bgs4∆::ura4^+^ Pbgs4^+^::GFP-bgs4^+^-leu1^+^ rlc1-tdTomato-natMX6 ura4? leu1-32 his3-D1? ade6?* | Fig 4G |
| JW6731 | *rlc1-tdTomato-natMX6 trs120-3GFP-kanMX6 ade6-M210 leu1-32 ura4-D18* | Fig 5, A-D |
| JW7318 | *rlc1-tdTomato-natMX6 trs120-3GFP-kanMX6 ync13Δ::kanMX6 ade6*  *leu1-32 ura4-D18* | Fig 5, A-D |
| JW9461 | *rlc1-tdTomato-natMX6 trs120-3GFP-kanMX6 rga7Δ::kanMX6*  *ade6-M210 leu1-32 ura4-D18* | Fig 5, A and B |
| JW9462 | *rlc1-tdTomato-natMX6 trs120-3GFP-kanMX6 rng10Δ::hphMX6*  *ade6-M210 leu1-32 ura4-D18* | Fig 5, A-D |
| JW9459 | *rlc1-tdTomato-natMX6 trs120-3GFP-kanMX6 rng10Δ::hphMX6*  *rga7Δ::kanMX6 ade6-M210 leu1-32 ura4-D18* | Fig 5, A and B |
| JW4028 | *h^-^ rga7Δ::kanMX6 ade6-M210 leu1-32 ura4-D18* | Fig 6, A-D |
| JW10431 | *h^+^ pmo25-GBP-hphMX6 ade6-M210 leu1-32 ura4-D18* | Fig 6, A-D |
| JW5752 | *h^-^ kanMX6-3nmt1-mECitrine-ync13 ade6-M210 leu1-32 ura4-D18* | Fig 6, A-D |
| JW10450 | *3nmt1-mECitrine-ync13 rga7Δ::natMX6 ade6-M210 leu1-32 ura4-D18* | Fig 6, A-D |
| JW10449 | *pmo25-GBP-hphMX6 rga7Δ::natMX6 ade6-M210 leu1-32 ura4-D18* | Fig 6, A-D |
| JW10435 | *pmo25-GBP-hphMX6 kanMX6-3nmt1-mECitrine-ync13 ade6-M210*  *leu1-32 ura4-D18* | Fig 6, A-D |
| JW10448 | *pmo25-GBP-hphMX6 kanMX6-3nmt1-mECitrine-ync13 rga7Δ::natMX6*  *ade6-M210 leu1-32 ura4-D18* | Fig 6, A-D |
| JW6153 | *bgs4∆::ura4^+^ Pbgs4^+^::GFP-bgs4^+^-leu1^+^ rlc1-tdTomato-natMX6*  *leu1-32 ura4-D18 his3-D1? ade6?* | Fig 7, A and C |
| JW8831 | *ync13Δ::kanMX6 ade6 leu1-32 ura4-D18* | Fig 7, B and C |
| JW7638 | *rga7-mCherry-natMX6 tom20-GBP-hphMX6 ade6-M210 leu1-32*  *ura4-D18* | Fig S1A |
| JW9005 | *rng10-mCherry-natMX6 tom20-GBP-hphMX6 ade6 leu1-32 ura4-D18* | Fig S1B |
| JW9465 | *kanMX6-3nmt1-mECitrine-ync13 tom20-GBP-hphMX6 ade6-M210*  *leu1-32 ura4-D18* | Fig S1C |
| JW9490 | *sec1-tdTomato-natMX6 tom20-GBP-hphMX6 ade6-M210 leu1-32*  *ura4-D18* | Fig S1D |
| JW6969 | *tom20-GBP-hphMX6 bgs4∆::ura4^+^ Pbgs4^+^::RFP-bgs4^+^-leu1^+^*  *ade6-M210 leu1-32 ura4-D18 his3-D1?* | Fig S1E |
| JW7608 | *ags1∆ 3’UTR_ags1_^+^::ags1^+^-Cherry:leu1^+^:ura4^+^ tom20-GBP-hphMX6*  *ade6-M210 leu1-32 ura4-D18* | Fig S1F |
| JW8868 | *ync13-tdTomato-natMX6 tom20-GBP-hphMX6 ade6-210 leu1-32*  *ura4-D18* | Fig S1G |
| JW8947 | *rng10-mEGFP-kanMX6 tom20-GBP-hphMX6 ade6-M210 leu1-32*  *ura4-D18* | Fig S1H |
| JW9314 | *tom20-GBP-hphMX6 rga7-mEGFP-kanMX6 ade6-M210 leu1-32*  *ura4-D18 his3-D1?* | Fig S1I |
| JW9475 | *kanMX6-3nmt1-mECitrine-ync13 rga7-mCherry–natMX6*  *tom20-GBP-hphMX6 ade6-M210 leu1-32 ura4-D18* | Fig S2A |
| JW9477 | *kanMX6-3nmt1-mECitrine-ync13 rng10-mCherry-natMX6*  *tom20-GBP-hphMX6 ade6 leu1-32 ura4-D18* | Fig S2A |
| JW9488 | *sec1-tdTomato-natMX6 kanMX6-3nmt1-mECitrine-ync13*  *tom20-GBP-hphMX6 ade6-M210 leu1-32 ura4-D18* | Fig S2B |
| JW9500 | *sec3-mCherry-natMX6 kanMX6-3nmt1-mECitrine-ync13*  *tom20-GBP-hphMX6 ade6-M210 leu1-32 ura4-D18* | Fig S2C |
| JW9473 | *kanMX6-3nmt1-mECitrine-ync13 ede1-mCherry-natMX6*  *tom20-GBP-hphMX6 ade6-M210 leu1-32 ura4-D18* | Fig S2D |
| JW9478 | *kanMX6-3nmt1-mECitrine-ync13 fim1-mCherry-natMX6*  *tom20-GBP-hphMX6 ade6-M210 leu1-32 ura4-D18* | Fig S2E |
| JW9495 | *clc1-mCherry-natMX6 kanMX6-3nmt1-mECitrine-ync13*  *tom20-GBP-hphMX6 his3? ade6-M21 ? leu1-32 ura4-D18* | Fig S2F |
| JW10433 | *rng10Δ::hphMX6 tom20-GBP-hphMX6 kanMX6-3nmt1-mECitrine-ync13*  *sec1-tdTomato-natMX6 ade6-M210 leu1-32 ura4-D18* | Fig S2G |
| JW9450 | *rng10-mEGFP-kanMX6 tom20-GBP-hphMX6 sec3-tdTomato-hphMX6*  *ade6-M210 leu1-32 ura4-D18* | Fig S3A |
| JW10399 | *tom20-GBP-hphMX6 rga7-mEGFP-kanMX6 sec3-tdTomato-hphMX6*  *ade6-210 leu1-32 ura4-D18* | Fig S3A |
| JW9498 | *bgs4∆::ura4^+^ Pbgs4^+^::RFP-bgs4^+^-leu1^+^ kanMX6-3nmt1-mECitrine-*  *ync13 tom20-GBP-hphMX6 ade6-M210 leu1-32 ura4-D18* | Fig S3B |
| JW9471 | *ags1∆ 3’UTR_ags1_^+^::ags1^+^-Cherry:leu1^+^:ura4^+^ kanMX6-3nmt1-mECitrine-*  *ync13 tom20-GBP-hphMX6 ade6-M210 leu1-32 ura4-D18* | Fig S3B |
| JW9436 | *bgs1∆::ura4^+^ Pbgs1^+^-tdTomato-bgs1^+^:leu1^+^ rga7-mEGFP-kanMX6*  *tom20-GBP-hphMX6 leu1-32 ura4-D18 his3-D1? ade6-M210?* | Fig S3C |
| JW9371 | *bgs1∆::ura4^+^ Pbgs1^+^-tdTomato-bgs1^+^:leu1^+^ rng10-mEGFP-kanMX6*  *tom20-GBP-hphMX6 leu1-32 ura4-D18 his3-D1? ade6 M210 ?* | Fig S3C |
| JW8890 | *ync13-tdTomato-natMX6 tom20-GBP-hphMX6 rga7FBD-mEGFP-*  *kanMX6 ade6 leu1-32 ura4-D18* | Fig S4B |
| JW8986 | *ync13-tdTomato-natMX6 tom20-GBP-hphMX6 rga7::kanMX6*  *GFP-rga7(∆F-BAR):leu1^+^ ade6-M210 leu1-32 ura4-D18* | Fig S4C |
| JW8942 | *ync13-tdTomato-natMX6 tom20-GBP-hphMX6*  *rng10(1-200)-mEGFP-kanMX6 ade6-M210 leu1-32 ura4-D18* | Fig S4D |
| JW8949 | *ync13-tdTomato-natMX6 tom20-GBP-hphMX6 kanMX6-Prng10-*  *mECitrine-rng10(201-1038) ade6-M210 leu1-32 ura4-D18* | Fig S4E |
| JW8952 | *ync13-tdTomato-natMX6 tom20-GBP-hphMX6 rng10(1-750)-mEGFP-*  *kanMX6 ade6-210 leu1-32 ura4-D18* | Fig S4F |
| JW8989 | *ync13-tdTomato-natMX6 tom20-GBP-hphMX6 kanMX6-Prng10-*  *mECitrine-rng10(751-1038) ade6-210 leu1-32 ura4-D18* | Fig S4G |
| JW10442 | *rga7Δ::kanMX6 kanMX6-Prng10-mECitrine-rng10(751-1038)*  *tom20-GBP-hphMX6 ync13-tdTomato-natMX6 ade6-210 leu1-32*  *ura4-D18* | Fig S4H |
| JW5969 | *ync13-mECitrine-kanMX6 rlc1-mCherry-natMX6 ade6-210 leu1-32*  *ura4-D18* | Fig S6A |
| JW8895 | *ync13-mECitrine-kanMX6 rlc1-mCherry-natMX6 rga7Δ::natMX6*  *ade6-M210 leu1-32 ura4-D18* | Fig S6A |
| JW8913 | *ync13-mECitrine-kanMX6 rlc1-mCherry-natMX6 rng10Δ::hphMX6*  *ade6-210 leu1-32 ura4-D18* | Fig S6A |
| JW3660 | *h^-^ rga7-mEGFP-kanMX6 ade6 leu1-32 ura4-D18* | Fig S6B |
| JW6063 | *h^-^ ync13Δ::kanMX6 rlc1-tdTomato-natMX6 rga7-mEGFP-kanMX6*  *ade6 leu1-32 ura4-D18* | Fig S6B |
| JW9470 | *kanMX6-3nmt1-ync13 rlc1-tdTomato-natMX6 rga7-mEGFP-kanMX6*  *ade6-M210 leu1-32 ura4-D18* | Fig S6B |
| JW5675 | *h^-^ rng10-mEGFP-kanMX6 ade6-M210 leu1-32 ura4-D18* | Fig S6C |
| JW9483 | *rng10-mEGFP-kanMX6 rlc1-tdTomato-natMX6 ync13Δ::kanMX6*  *ade6 leu1-32 ura4-D18* | Fig S6C |
| JW9434 | *rng10-mEGFP-kanMX6 kanMX6-3nmt1-ync13 ade6-M210 leu1-32*  *ura4-D18* | Fig S6C |
| JW6153 | *bgs4∆::ura4^+^ Pbgs4^+^::GFP-bgs4^+^-leu1^+^ rlc1-tdTomato-natMX6*  *leu1-32 ura4-D18  his3-D1? ade6?* | Fig S6, D and E |
| JW9566 | *sec1-M2-his5^+^-kanMX6 bgs4∆::ura4^+^ Pbgs4^+^::GFP-bgs4^+^-leu1^+^*  *rlc1-tdTomato-natMX6 leu1-32 ura4 his3? ade6?* | Fig S6D |
| JW9563 | *bgs4∆::ura4^+^ Pbgs4^+^::GFP-bgs4^+^-leu1^+^ rlc1-tdTomato-natMX6*  *trs120-ts1-his5^+^-kanMX6 his5? leu1-32 ura4* *ade6?* | Fig S6E |
| JW3693 | *rga7-mEGFP-kanMX6 rlc1-tdTomato-natMX6 ade6-M210 leu1-32*  *ura4-D18* | Fig S6F |
| JW9673 | *cwg1-1 rga7-mEGFP-kanMX6 rlc1-tdTomato-natMX6 ade6-210?*  *leu1-32? ura4-D18* | Fig S6F |
| JW5899 | *rng10-mEGFP-kanMX6 rlc1-tdTomato-natMX6 ade6-M210 leu1-32*  *ura4-D18* | Fig S6G |
| JW9671 | *cwg1-1 rng10-mEGFP-kanMX6 rlc1-tdTomato-natMX6 ade6-210?*  *leu1-32 ura4-D18?* | Fig S6G |
| JW9672 | *cwg1-2 rng10-mEGFP-kanMX6 rlc1-tdTomato-natMX6 ade6-M210*  *leu1-32 ura4* | Fig S6G |
| JW5249 | *GFP-bgs1-leu1^+^ bgs1Δ::ura4^+^ rlc1-tdTomato-natMX6 ade6-M210*  *leu1-32 ura4-D18* | Fig S7A |
| JW6108 | *GFP-bgs1-leu1^+^ bgs1Δ::ura4^+^ rlc1-tdTomato-natMX6 rng10Δ::kanMX6*  *ade6-210 leu1-32 ura4-D18* | Fig S7B |
| JW8128 | *GFP-bgs1-leu1^+^ bgs1Δ::ura4^+^ rlc1-tdTomato-natMX6 ync13-19-his5^+^*  *-kanMX6 ade6-M210 leu1-32? ura4* | Fig S7C |
| JW9052 | *GFP-bgs1-leu1^+^ bgs1Δ::ura4^+^ ync13-19-his5^+^-kanMX6 rng10Δ::*  *kanMX6 ade6-210 leu1-32 ura4* | Fig S7D |
| JW6731 | *rlc1-tdTomato-natMX6 trs120-3GFP-kanMX6 ade6-M210 leu1-32*  *ura4-D18* | Fig S8A |
| JW7318 | *rlc1-tdTomato-natMX6 trs120-3GFP-kanMX6 ync13Δ::kanMX6 ade6*  *leu1-32 ura4-D18* | Fig S8A |
| JW9461 | *rlc1-tdTomato-natMX6 trs120-3GFP-kanMX6 rga7Δ::kanMX6*  *ade6-M210 leu1-32 ura4-D18* | Fig S8A |
| JW9462 | *rlc1-tdTomato-natMX6 trs120-3GFP-kanMX6 rng10Δ::hphMX6*  *ade6-M210 leu1-32 ura4-D18* | Fig S8A |
| JW9459 | *rlc1-tdTomato-natMX6 trs120-3GFP-kanMX6 rng10Δ::hphMX6*  *rga7Δ::kanMX6 ade6-M210 leu1-32 ura4-D18* | Fig S8A |
| JW9493 | *kanMX6-Pypt3-tdTomato-ypt3 kanMX6-3nmt1-mECitrine-ync13*  *ade6-M210 leu1-32 ura4-D18* | Fig S8B |
| TP150 | *h^-^ leu1-32 SM902* | S1 Table |
| JW6731 | *rlc1-tdTomato-natMX6 trs120-3GFP-kanMX6 ade6-M210 leu1-32*  *ura4-D18* | S1 Video |
| JW9462 | *rlc1-tdTomato-natMX6 trs120-3GFP-kanMX6 rng10Δ::hphMX6*  *ade6-M210 leu1-32 ura4-D18* | S2 Video |
| JW7318 | *rlc1-tdTomato-natMX6 trs120-3GFP-kanMX6 ync13Δ::kanMX6 ade6*  *leu1-32 ura4-D18* | S3 Video |
