# Supplementary material for: The Ync13–Rga7–Rng10 complex selectively coordinates secretory vesicle trafficking and secondary septum formation during cytokinesis
Source: PLoS Biol. 2025 Oct 27;23(10):e3003466. doi: 10.1371/journal.pbio.3003466 (PMC12574955; doi:10.1371/journal.pbio.3003466)
Supplement: S1 Raw images — Each of the full-size, uncropped, and labeled Western blot or SDS–PAGE gel is shown for the indicated figure panels. The relevant protein is labeled and indicated with an arrowhead. Irrelevant lanes, which are not included in figures, are labeled on the top. (PDF) [file pbio.3003466.s014.pdf]

Uncropped Western blots/SDS-PAGE gels for figures shown in:

The Ync13-Rga7-Rng10 complex selectively coordinates secretory vesicle trafficking and secondary septum formation during cytokinesis

Sha Zhang[1¶], Davinder Singh[1¶], Yi-Hua Zhu[1], Katherine J. Zhang[1], Alejandro Melero[2], Sophie G. Martin[2,3], and Jian-Qiu Wu[1*]

Each of the full size, uncropped and labeled western blot or SDS-PAGE gel is shown for the indicated figure panels. The relevant protein is labeled and indicated with an arrowhead. Irrelevant lanes, which are not included in figures, are labeled on the top.

Figure 2A-GFP

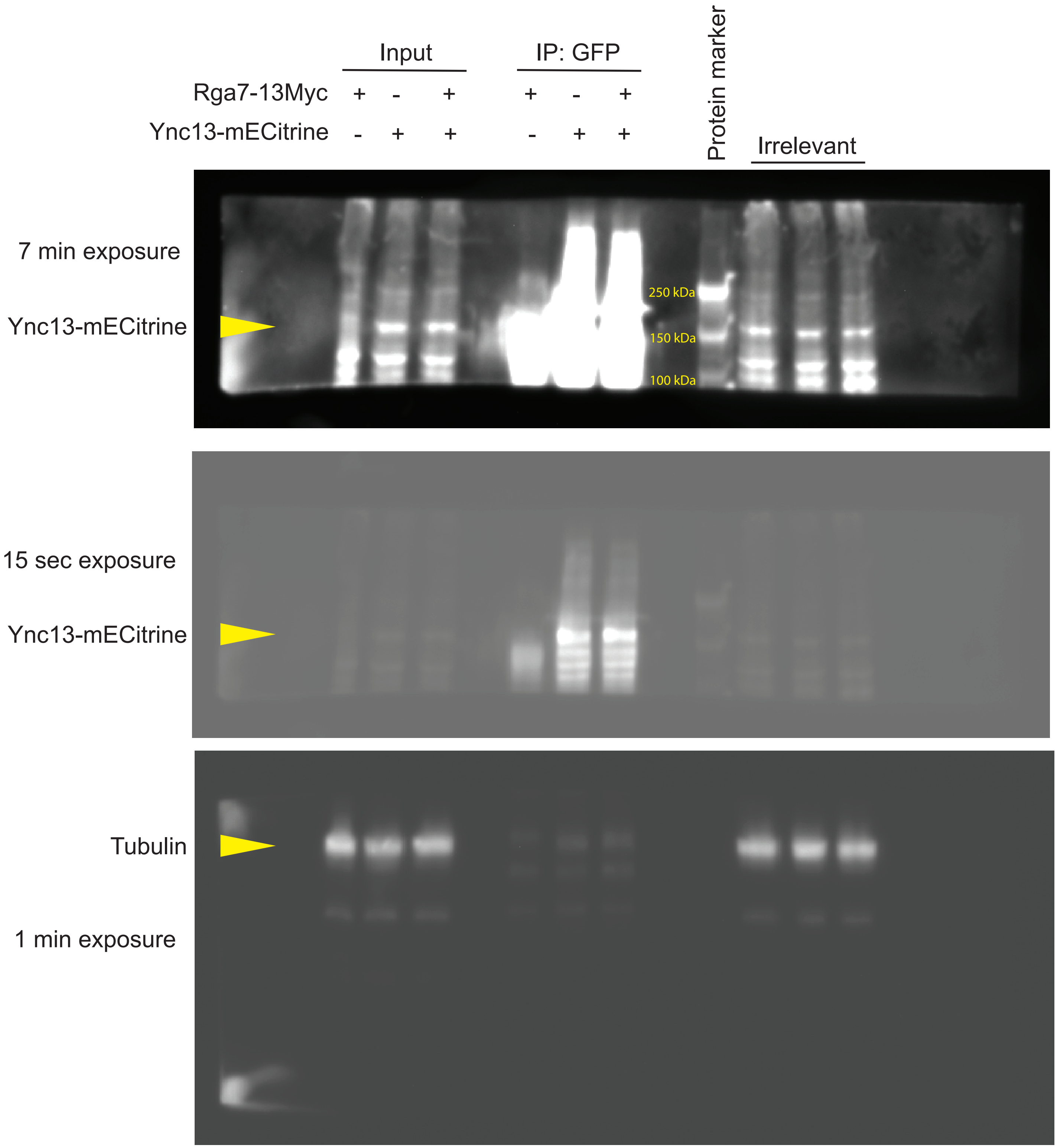

After transfer, membrane was cut into 2 pieces, the top half of membrane was incubated with anti-GFP and the lower half with anti-tubulin antibody TAT1. The tubulin bands are not shown in the main Figure.

Figure 2A-Myc

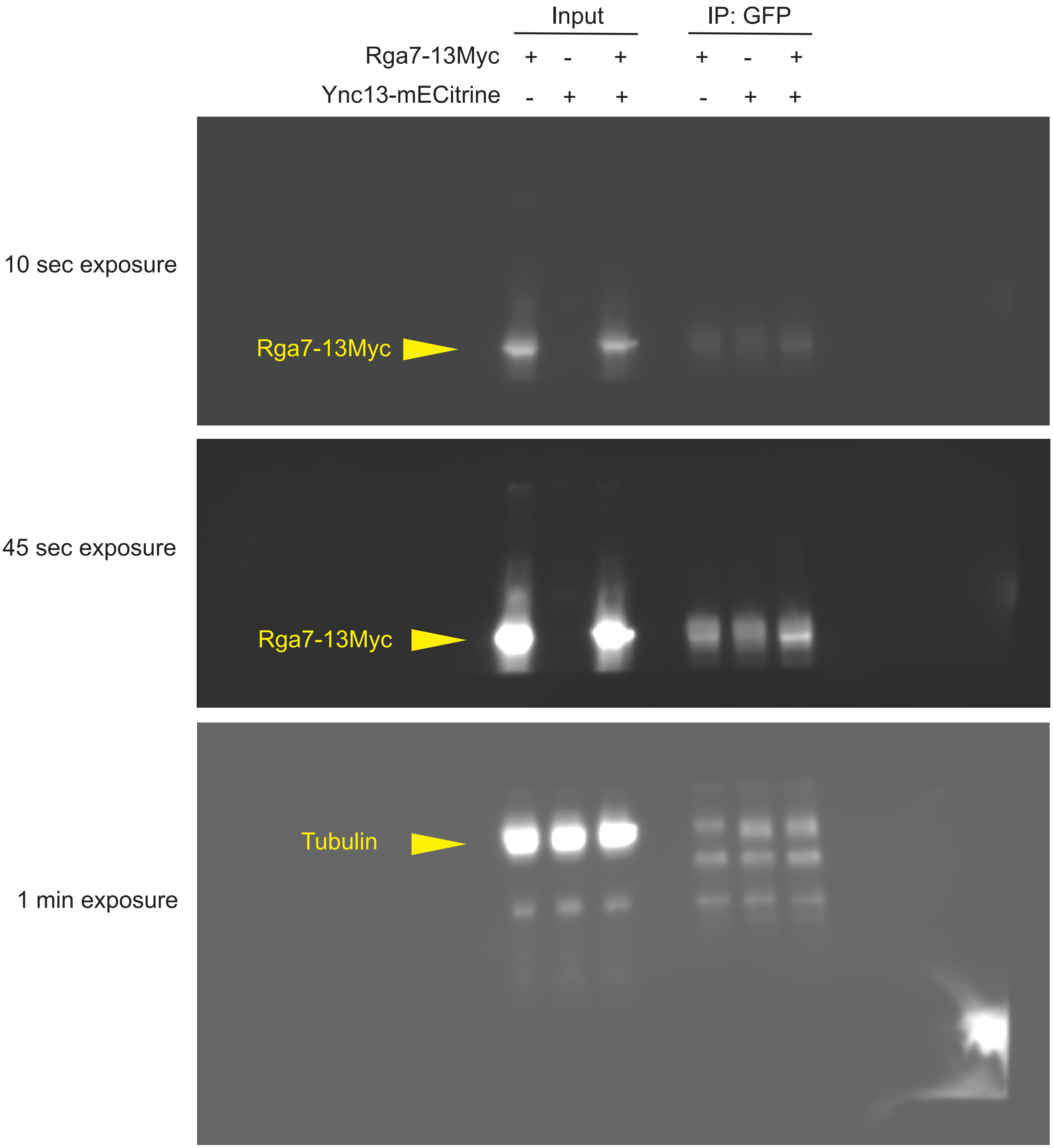

After transfer, membrane was cut into 2 pieces, the top half of membrane was incubated with anti-Myc and the lower half with anti-tubulin antibody TAT1. The tubulin bands are not shown in the main Figure.

Figure 2B

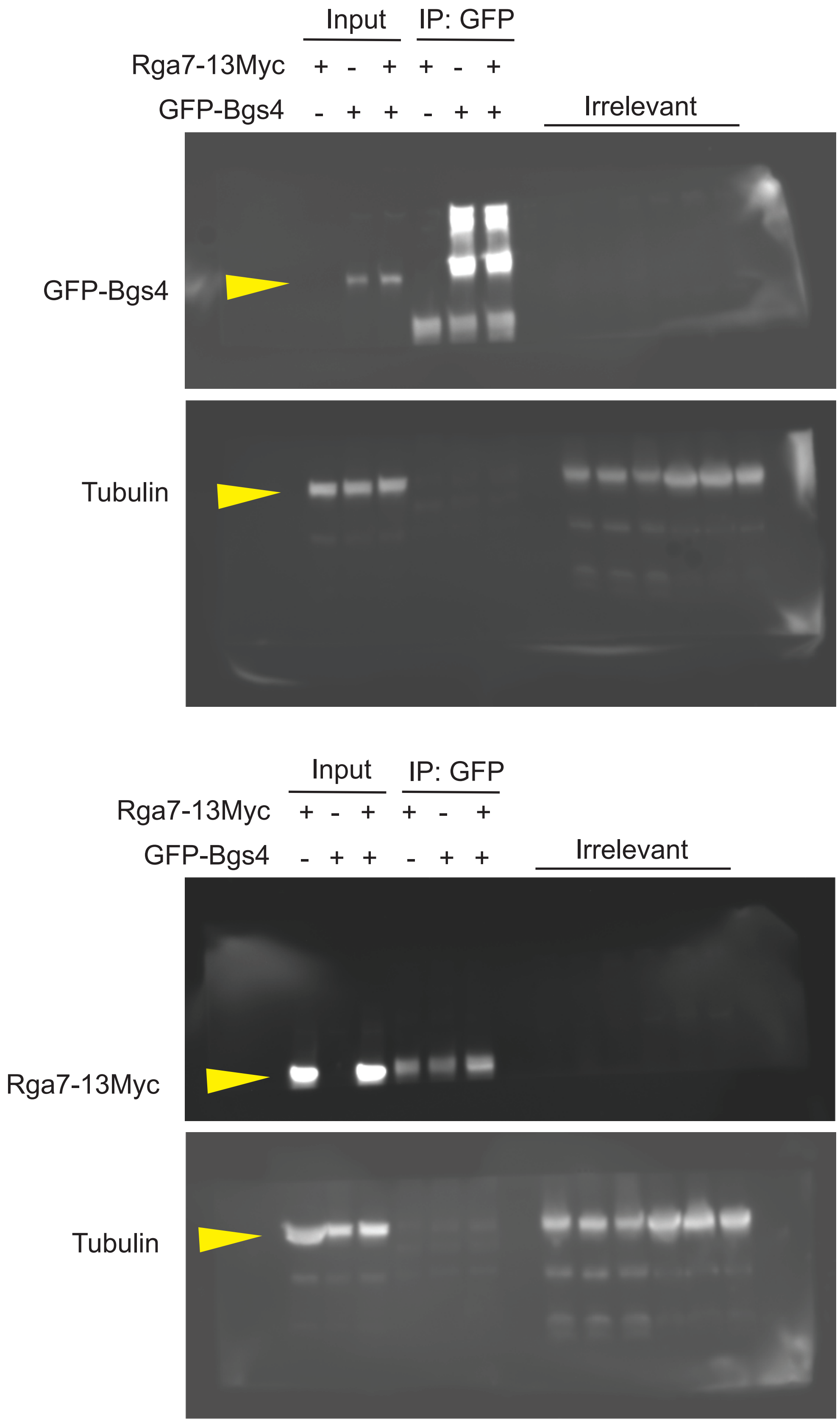

After transfer, the membrane was cut into 2 pieces, the top half of membrane was incubated with anti-GFP or anti-Myc the lower half with anti-tubulin antibody TAT1. The tubulin bands are not shown in the main Figure.

Figure 2C-left

*** Protein marker**

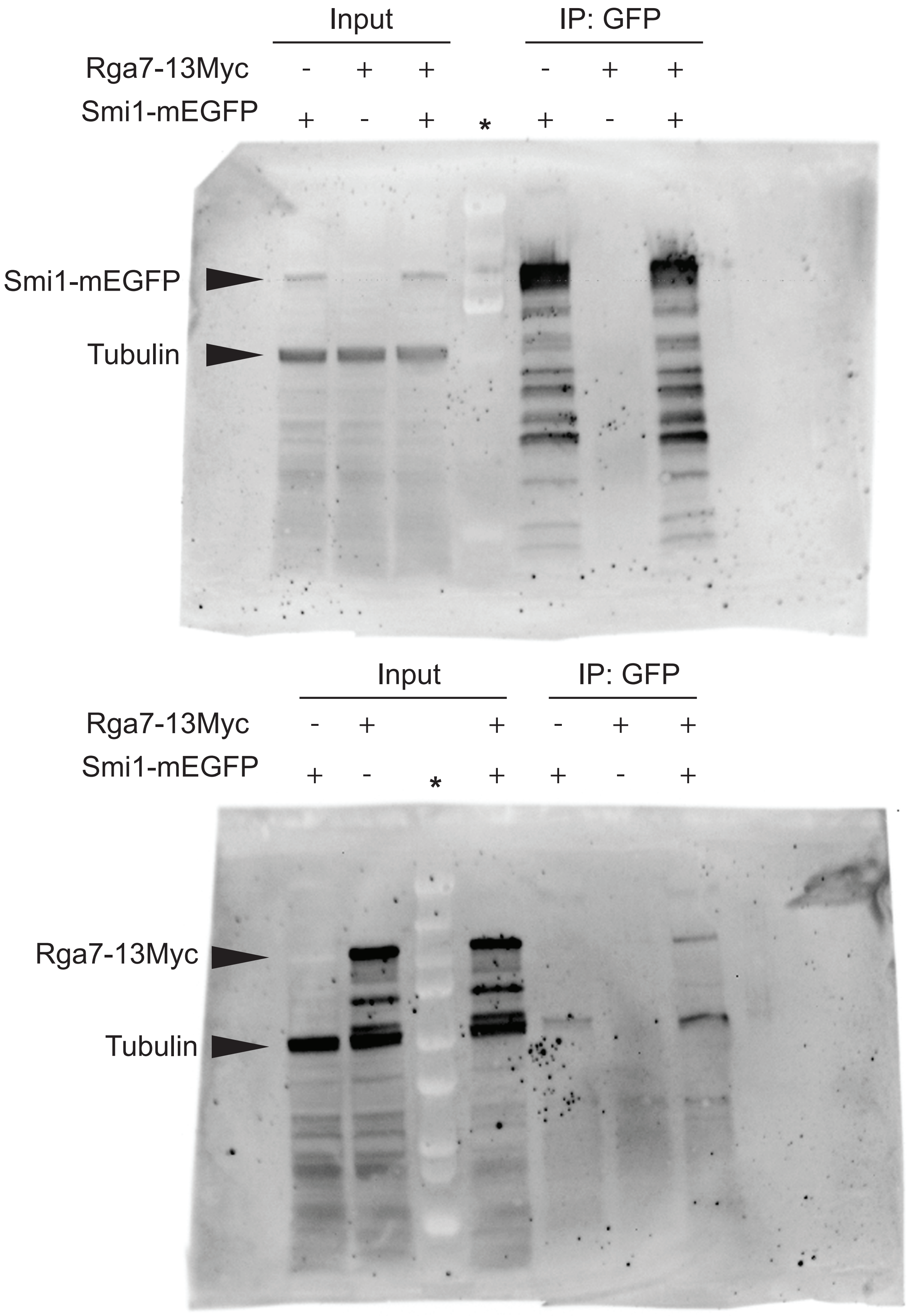

The tubulin bands are not shown in the main Figure.

Figure 2C-right

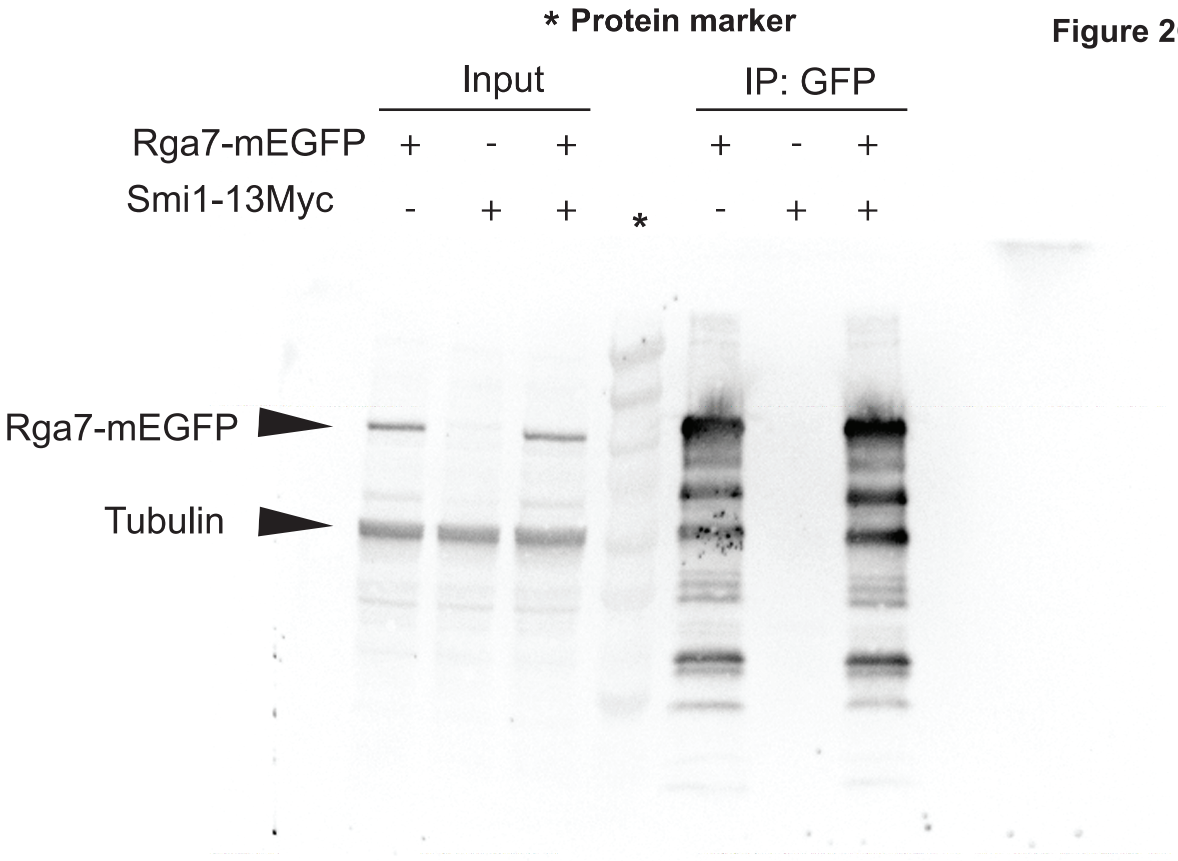

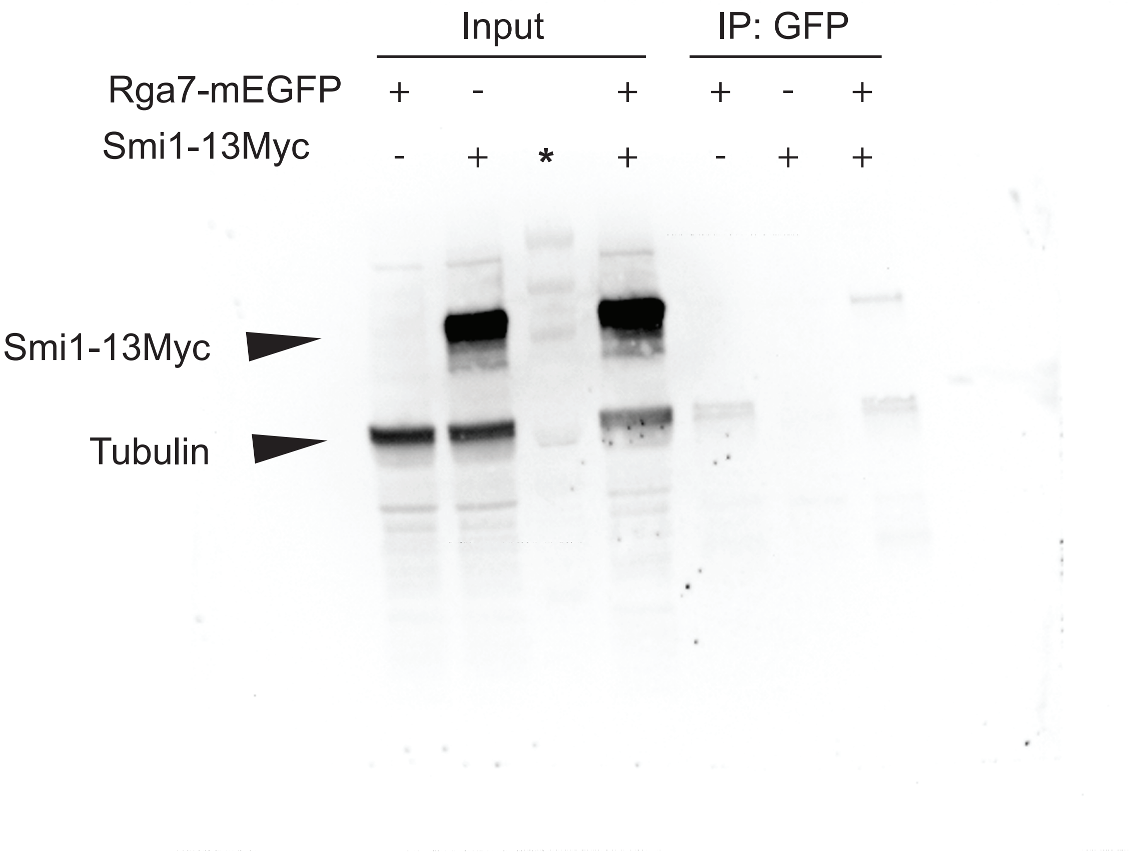

The tubulin bands are not shown in the main Figure.

Figure 2D

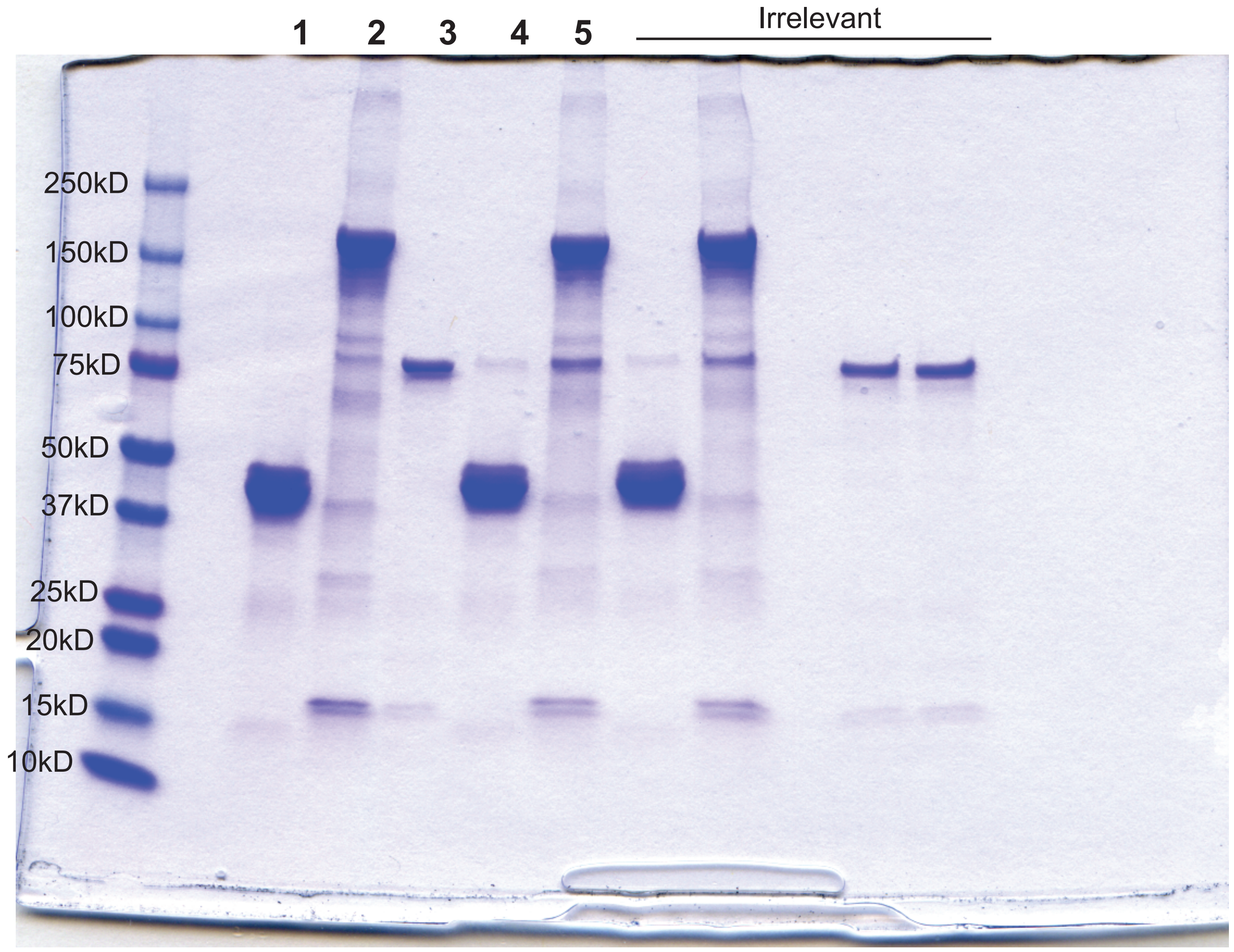

1. MBP-6His input
2. MBP-Ync13-6His input
3. Rga7-6His input
4. MBP-6His + Rga7-6His pull-down
5. MBP-Ync13-6His + Rga7-6His pulldown

Figure 2E

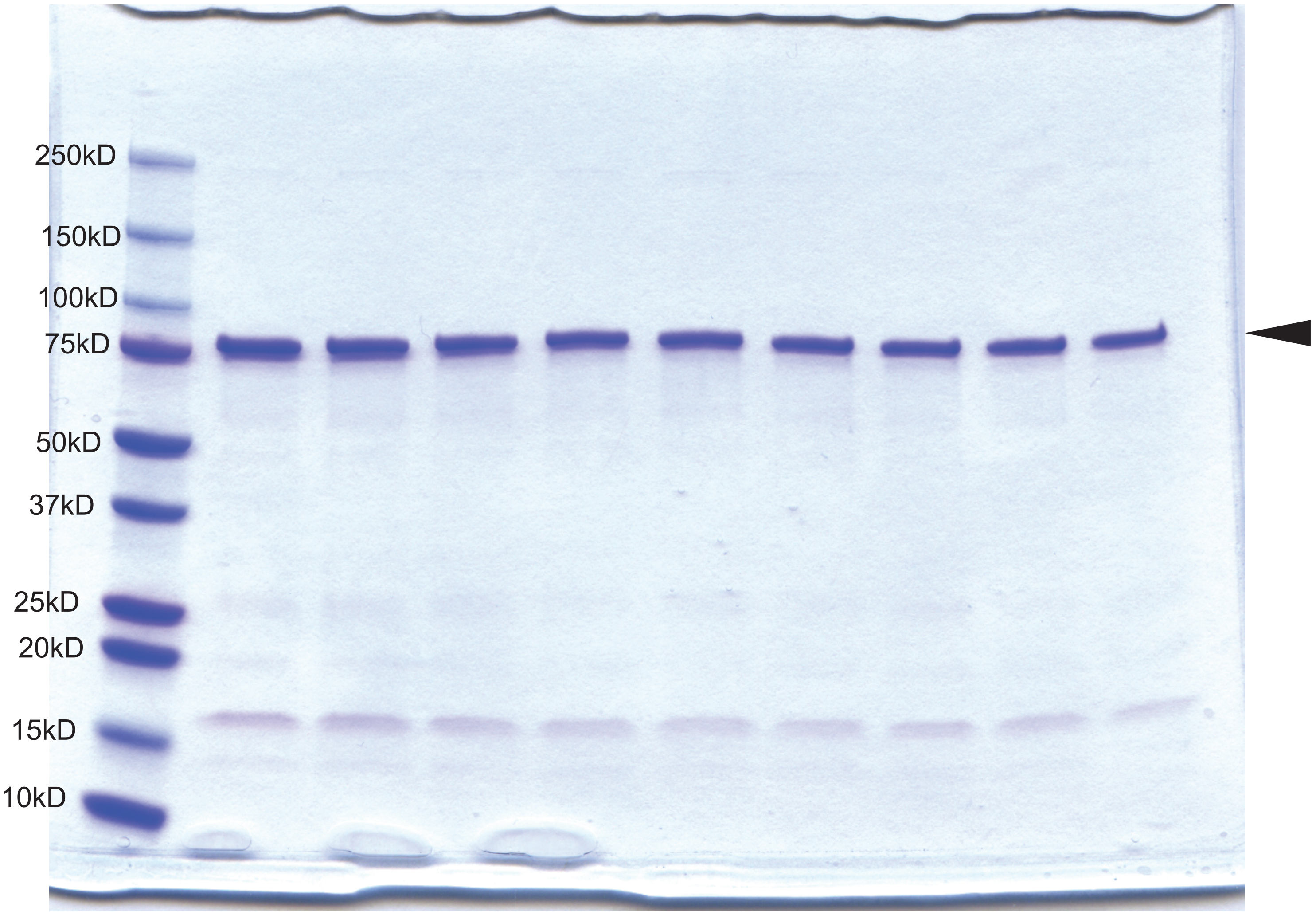

Rga7-6His 76.6 kDa

Figure 3C

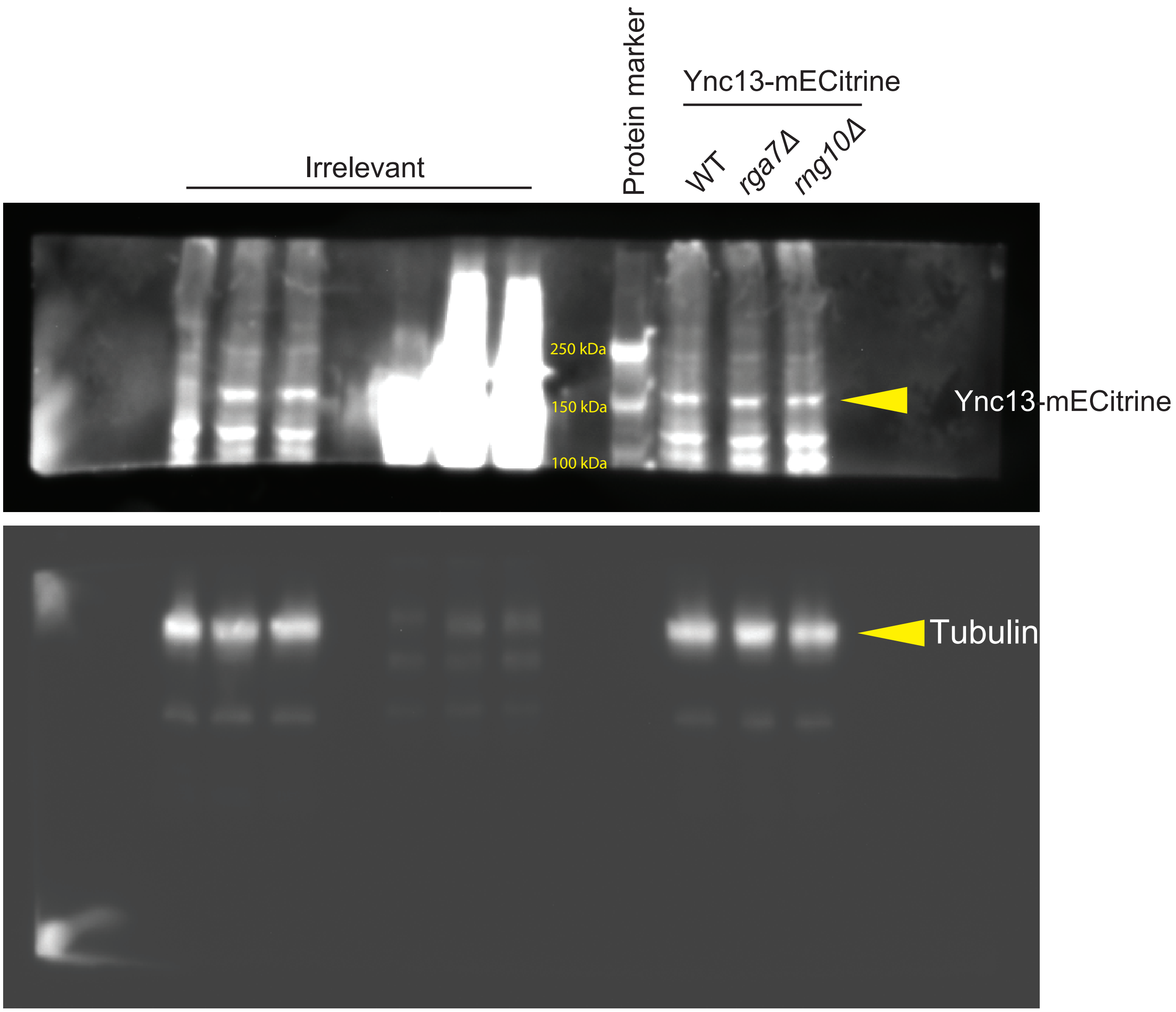

After transfer, the membrane was cut into 2 pieces, the top half of membrane was incubated with anti-GFP and the lower half with anti-tubulin antibody TAT1.

Figure 3D

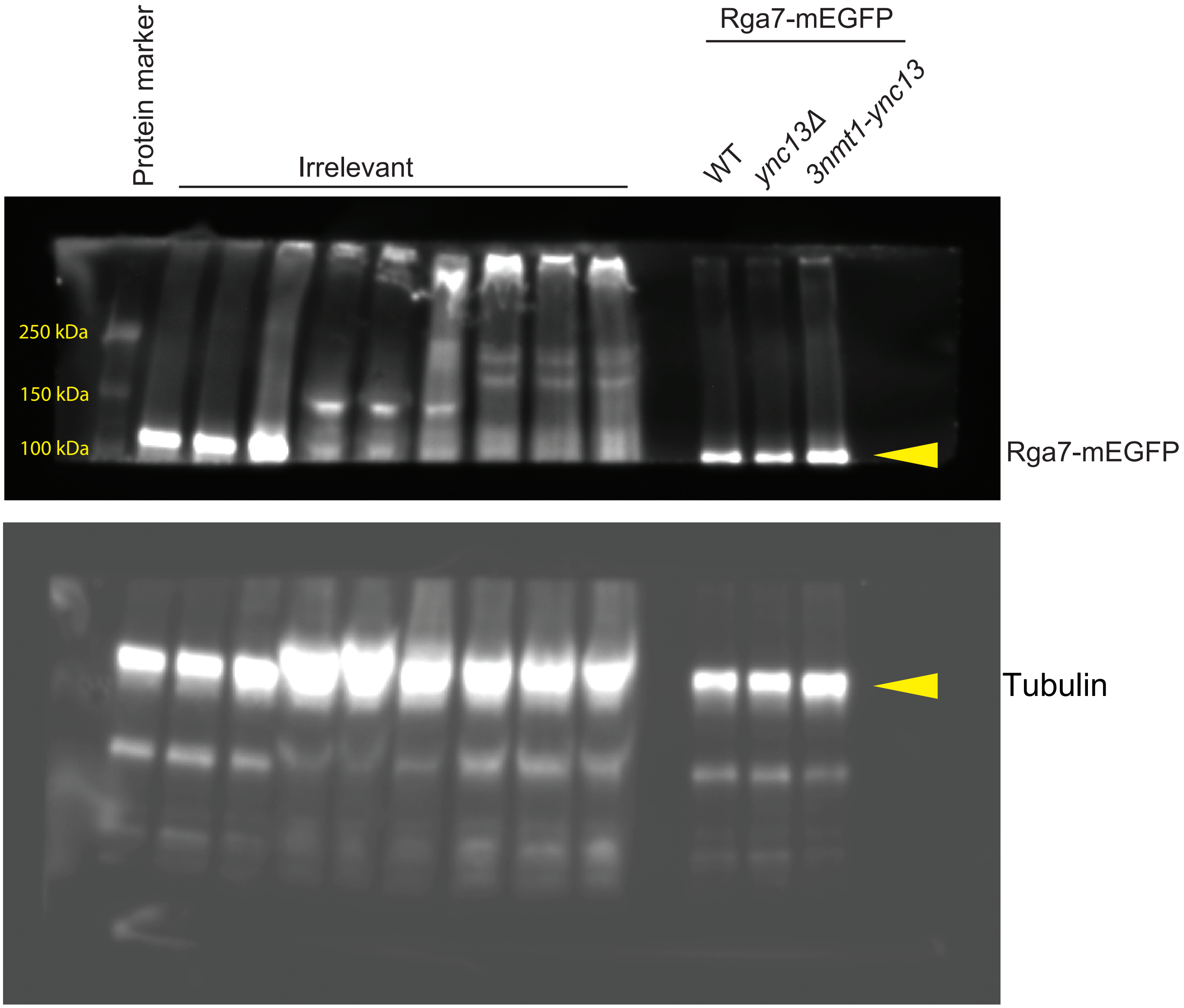

After transfer, the membrane was cut into 2 pieces, the top half of membrane was incubated with anti-GFP and the lower half with anti-tubulin antibody TAT1.

Figure S6C

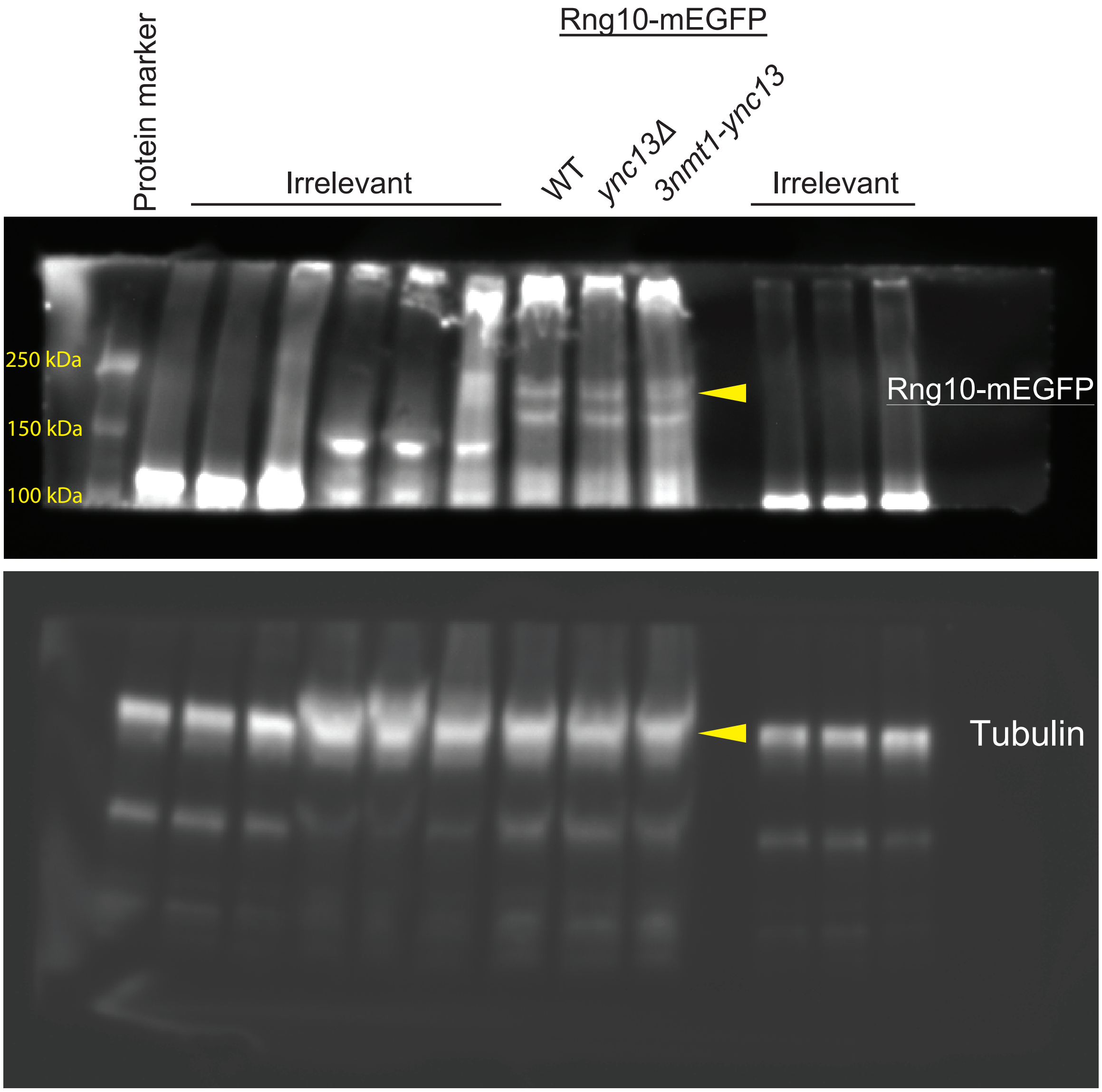

After transfer, the membrane was cut into 2 pieces, the top half of membrane was incubated with anti-GFP and the lower half with anti-tubulin antibody TAT1.
