# Supplementary material for: The Ync13–Rga7–Rng10 complex selectively coordinates secretory vesicle trafficking and secondary septum formation during cytokinesis
Source: PLoS Biol. 2025 Oct 27;23(10):e3003466. doi: 10.1371/journal.pbio.3003466 (PMC12574955; doi:10.1371/journal.pbio.3003466)
Supplement: Attachment — Submitted filename: 158271_1_rebuttal_3509987_t2jvbm.pdf [file pbio.3003466.s017.pdf]

Reviewer #1 (Evidence, reproducibility and clarity (Required)):

In this paper, the GFP-GBP system for mistargeting protein localization was used in fission yeast cells to discover new protein interactions involved in vesicular trafficking during cytokinesis. This approach uncovered a new association between the F-BAR protein Rga7 and its binding partner Rng10 with the Munc13 protein Ync13 at the cell division site. Additional associations were observed between Rga7-Rng10, Ync13 and the glucan synthases Ags1 and Bgs4, and the vesicle fusion protein Sec1. These interactions identified by the GFP-GBP system were further supported by co-immunoprecipitation experiments and by defining localization dependencies with live cell imaging in a variety of mutant strains. The imaging data are all of high quality and for the most part support the conclusions. However, in my opinion some of the interpretations are overstated, and the manuscript would benefit from providing additional mechanistic information. Major and minor recommendations are outlined below.

Major suggestions

1. *The co-IP data are interpreted to suggest that all the above-mentioned proteins form a single "big complex." However, as noted in the manuscript and reflected in the model, the multipass integral membrane proteins Bgs4 and Ags1 are embedded in the vesicle membrane and likely only indirectly associate with the scaffold Rga7-Rng10 via Ync13, without forming a 'complex'. One would expect the entirety of these vesicle contents to co-IP if the model is correct. The first paragraph of page 11 should be revised to more clearly reflect this scenario and to align with the proposed model.*

Response: We thank the reviewer for this thoughtful clarification. In the original manuscript, we stated "…indicating these proteins do interact or form big protein complexes… These results suggest that Rga7, Rng10, and Ync13 form a protein complex." We agree that our initial wording may have unintentionally implied that all proteins detected in co-IP experiments assemble into a single, large physical complex. As the reviewer correctly noticed, the multipass integral membrane proteins Bgs4 and Ags1 are embedded within vesicle membranes and are more likely to associate indirectly with the Rga7-Rng10-Ync13 complex, rather than being part of one unified protein complex. To avoid overinterpretation, we have modified the last sentence of the first paragraph on the original page 11 as below: "These results suggest that Rga7, Rng10, and Ync13 do form a protein complex, although maybe dynamic and not super stable (see Discussion). Our data indicate that Rga7 interacts with both Ync13 and Rng10 to form a module on the plasma membrane for targeting of the vesicles containing cargos such as glucan synthases Bgs4 and Ags1. However, these glucan synthases are multipass integral membrane embedded proteins and likely only indirectly associate with the module Rng10-Rga7-Ync13, without forming a big protein complex."

2. *Can Ync13 be artificially directed or tethered to the division site independently of Rga7-Rng10*

(e.g., via Imp2)? If so, can this rescue the phenotypes of rga7Δ cells? This experiment could clarify whether Ync13 is the key functional effector of the Rga7-Rng10 complex.

Response: We thank the reviewer for suggesting this interesting experiment. We agree that testing whether correctly localized Ync13 is sufficient to execute the division-site function of the Rga7–Rng10 complex would clarify its role. To test this, we artificially targeted Ync13 to the division site independently of Rga7 by tethering it to the scaffold protein Pmo25. Pmo25, an MO25 family protein, localizes to both the plasma membrane at the division site and the spindle pole body (mainly one of the SPBs) during mitosis and cytokinesis, enabling us to mislocalize Ync13 to these structures through GFP–GBP system. We did not use Imp2 because its localization pattern (mainly to the contractile ring [1, 2]) is different from Ync13. Microscopy revealed robust localization of Ync13 at the division site and the SPB in *rga7Δ* cells, and this tethered Ync13 persisted along the cleavage furrow throughout ring constriction. Importantly, enforced division-site localization of Ync13 significantly rescued the cytokinesis defects and cell lysis of *rga7Δ*. Consistently, growth assays on Phloxin B (PB) plate showed the elevated lysis/death in *rga7Δ* cells was rescued by Ync13 tethering to Pmo25-GBP. Together, these findings support that Ync13 is a key functional effector acting downstream of the Rga7–Rng10 scaffold at the division site. We have added these results in the new Figure 6 and associate text in the revised manuscript. We have also updated the model in Figure 8 to reflect this new result.

1. Demeter J, Sazer S. imp2, a new component of the actin ring in the fission yeast *Schizosaccharomyces pombe*. J Cell Biol. 1998;143(2):415-27. PubMed PMID: 9786952.
2. Martin-Garcia R, Coll PM, Perez P. F-BAR domain protein Rga7 collaborates with Cdc15 and Imp2 to ensure proper cytokinesis in fission yeast. J Cell Sci. 2014;127(Pt 19):4146-58. Epub 2014/07/24. doi: 10.1242/jcs.146233. PubMed PMID: 25052092.

3. *The authors should consider structural or computational modeling of the proposed Rga7-Rng10-Ync13 complex. Such analysis could offer insight into how these components interact and strengthen the proposed model.*

Response: We thank the reviewer for this valuable suggestion. Following the recommendation, we performed structural modeling of the Rga7–Rng10–Ync13 complex using AlphaFold3. Our previous work demonstrated that the F-BAR protein Rga7 forms a stable dimer and its F-BAR domain binds the C-terminal (aa751–1038) region of Rng10 [3]. Based on these findings, we constructed an input model consisting of two full-length Rga7 subunits, two Rng10(751–1038) subunits, and one full-length Ync13. The predicted structure revealed a modular organization in which Rng10(751–1038) associated strongly with the F-BAR domain of the Rga7 dimer, consistent with our prior biochemical data [3]. In addition, the model suggested that Ync13 interacted with the GAP domain of Rga7, positioning Ync13 in close proximity to the Rga7–Rng10 interface (Fig. S5, A, B, D and F). Further domain specific predictions confirmed the interactions between Rga7-GAP and Ync13 N-terminus (pTM: 0.63, ipTM: 0.64), two Rga7 F-

BARs (pTM: 0.74, ipTM: 0.71), as well as Rga7 F-BAR and Rng10(751–1038) (pTM: 0.56, ipTM: 0.78) (Fig. S5, C-F). Overlay analyses revealed that the interacting domains align well with the structure of whole complex as the root mean square differences (RMSDs) are <1 Å for all predictions. In particular, Rga7-GAP domain interacting with Ync13 N-terminus aligned perfectly with predicted structure of whole complex (Fig. S5, A and B). This arrangement provides a structural framework for how the Rga7 dimer, through direct interactions with Rng10 and Ync13, could function as a scaffold to recruit and stabilize these two proteins. Together, these modeling results support our experimental findings and provide a mechanistic rationale for the cooperative function of Rga7, Rng10, and Ync13 at the division site.

3. Liu Y, McDonald NA, Naegele SM, Gould KL, Wu J-Q. The F-BAR domain of Rga7 relies on a cooperative mechanism of membrane binding with a partner protein during fission yeast cytokinesis. Cell Rep. 2019;26(10):2540-8.e4. doi: 10.1016/j.celrep.2019.01.112. PubMed PMID: 30840879; PubMed Central PMCID: PMCPMC6425953.

Minor text edits

1. *Define "SIN" in the discussion section for clarity.*

Response: We defined the SIN pathway in the Discussion section as suggested: "At low restrictive temperatures, the lethality of mutant *sid2,* the most downstream kinase in the Septation Initiation Network, is partially rescued by upregulating Rho1. Thus, it has been suggested that the Septation Initiation Network activates Rho1, which in turn activates the glucan synthases [4]."

4. Alcaide-Gavilán M, Lahoz A, Daga RR, Jimenez J. Feedback regulation of SIN by Etd1 and Rho1 in fission yeast. Genetics. 2014;196(2):455-70. Epub 2013/12/18. doi: 10.1534/genetics.113.155218. PubMed PMID: 24336750; PubMed Central PMCID: PMCPMC3914619.

2. *Figure S3, the protein schematics should start at residue "1" and not "0".*

Response: We apologize for the mistake. The schematics in revised figure (now Figure S4A) have been corrected to start at residue 1.

3. *Mass spectrometry data referenced in the text are not provided in the manuscript.*

Response: We apologize for the omission. The mass spectrometry data are now shown in Table S1.

4. *In Figure 4A. The Ags1 rim localization does not appear decreased as the authors claim.*

Response:
After examining the data again, we agree with the reviewer's assessment. So, we reworded the

sentence as the following: “We also found that in *ync13Δ* cells, the Bgs4 intensity at the rim of the septum was much lower than in WT after ring constriction (Fig. 4B).”

5. *On page 13: "both Rga7 and Rng10 can mistarget Trs120 to mitochondria."*

Response: Thank you. The typo “mistargeting” has been corrected to “mistarget”.

Minor figure edits

1. *Consider inverting single-channel images to display fluorescence on a white background, which would improve visual clarity.*

Response: We appreciate the reviewer’s suggestion. However, we have chosen to retain the original display format with fluorescence shown in a black background, to be consistent with our (and some others’) previous publications. We believe this format preserves clarity while allowing easier comparison with the previously published works.

2. *The Figure 1 legend should describe the experimental setup rather than restating conclusions.*

Response: We thank the reviewer for this helpful suggestion. The Figure 1 legend has been revised to describe the experimental setup and imaging conditions rather than summarizing conclusions as the following:

Fig. 1. Physical interactions among the key cytokinetic proteins in plasma membrane deposition and septum formation revealed by ectopic mistargeting to mitochondria by Tom20-GBP. Arrowheads mark examples of colocalization at mitochondria. **(A)** Ync13 colocalizes with Rga7 and Rng10 at cell tips and the division site. **(B-F)** Tom20-GBP can ectopically mistarget Rga7/Rng10-mEGFP and their interacting partners tagged with tdTomato/RFP/mCherry to mitochondria. Tom20–GBP was used to recruit mEGFP-tagged Rga7 or Rng10 to mitochondria, and colocalization was assessed with tdTomato/RFP/mCherry-tagged candidate binding partners. Cells were grown at 25ºC in YE5S + 1.2 M sorbitol medium for ~36 to 48 h and then were washed with YE5S without sorbitol and grown in YE5S for 4 h before imaging. **(B)** Rga7/Rng10-Ync13. **(C)** Rga7/Rng10-Trs120. **(D)** Rga7/Rng10-Bgs4. **(E)** Rga7/Rng10-Ags1. **(F)** Rga7-Smi1. Bars, 5 µm.

3. *Reduce the number of arrows indicating co-localization in microscopy images; highlighting 1-2 representative examples is sufficient and less visually cluttered.*

Response: We appreciate the reviewer’s suggestion. We have revised the micrographs to reduce the number of arrowheads, highlighting several representative examples of co-localization per image. This improves clarity and reduces visual clutter while still guiding the reader to the key observations.

4. *Figure 3F, the scale bar is listed as 5 µm in the legend but it appears to my eye to be 2 µm.*

Response: We thank the reviewer for noticing this error. After rechecking the original imaging data, we have added a new 5 µm scale bar.

5. *The orientation of Bgs4/Smi1 should be inverted in the schematic within vesicles so that Smi1 is always on the cytoplasmic side.*

Response: We thank the reviewer for pointing out this error. The schematic has been corrected so that Bgs4 and Smi1 are oriented appropriately, with Smi1 consistently placed on the cytoplasmic side of vesicles because it does not have a transmembrane domain. The revised schematic is included in the updated Figure 8.

6. *Also in the schematic, Mid1 is not at the constricting CR and therefore needs to be removed.*

Response: Thank you for the suggestion. Mid1 has been removed from the model figure.

Reviewer #1 (Significance (Required):
From the data presented in the manuscript, it is proposed that Rga7 and Rng10 form a scaffold at the division site for delivery of exocytic vesicles marked by the TRAPPII complex but not the exocyst complex. Further, it is proposed that these vesicles deliver specifically the glucan synthases necessary for septation. Overall, this study builds on previous work from the Wu lab to clarify how the TRAPPII-decorated vesicles are specifically delivered to the cell division site, adding some new information about vesicle trafficking regulation during cytokinesis. It also provides new insight into the role of a F-BAR scaffold protein.

This paper will be of interest to those studying cytokinesis and also those studying mechanisms of intracellular trafficking.

Reviewer expertise: Cell division, signaling, membrane biology

Reviewer #2 (Evidence, reproducibility and clarity (Required)):

Summary:

This paper provides a comprehensive analysis of the roles of Rng10, Rga7, and Ync13 in cytokinesis using fission yeast as a model system. The authors demonstrate that Ync13/Rna7/Rng10 not only interact with each other but also associate with components of glucan synthases, which are essential for secondary septum formation but not for the primary septum. They further show that Ync13 is involved in exocytosis through its interaction with Sec1 and plays a role in membrane trafficking via interaction with the TRAPP-II complex. Collectively, their findings reveal a coordinated mechanism that ensures the timely formation of the secondary septum during cytokinesis, as deletion of these proteins disrupts septum formation and leads to cell lysis.

The conclusions drawn in this paper are well-supported by the data, with a clear methodology and robust statistical analyses that enhance reproducibility. However, I have the following major and minor comments:

Major Comments -

1) *The authors propose that Ync13, Rng10, and Rga7 interact to form a protein complex, supported by their mislocalization studies. While these findings are suggestive, additional co-immunoprecipitation (co-IP) data specifically demonstrating a direct interaction between Ync13 and Rng10 would strengthen the claim.*

Response: We thank the reviewer for this suggestion. The direct interaction between Rga7 with Rng10 has been already established and published by our group [3, 5]. Here we found that Rga7 and Ync13 directly interact by in vitro binding assay (Figure 2, D and E). While our current data do not suggest a direct physical interaction between Ync13 and Rng10, our mislocalization results and other data do provide strong support for their functional association. In particular, ectopic tethering of Ync13 to mitochondria recruits Rng10 to the same sites and vice versa (Figures. 1B and S2A). Additionally, division-site tethering of Ync13 by Pmo25-GBP rescues both the growth and cell-lysis phenotype of *rga7Δ* (Figure 6), consistent with the idea that Ync13 functions downstream of Rga7-Rng10 because Rga7 localization depends on Rng10 (Figure 8). Furthermore, our AlphaFold3 modeling predicts that Rng10 binds the BAR domain of Rga7, whereas Ync13 binds the GAP domain of Rga7, suggesting that Rng10 and Ync13 are positioned within the same complex through Rga7 without direct interaction (Figure S5).

The predicted lack of direct interaction between Ync13 and Rng10(751–1038) is supported by the experiment mentioned below to answer the minor question from the Reviewer 3. We tested the mistargeting of mECitrine-Rng10(751–1038) in *rga7Δ tom20-GBP* cells and found that Ync13-tdTomato could not be recruited to mitochondria (Figure S4H). This indicates

that Ync13 cannot interact with Rng10(751–1038) independently of Rga7, supporting our proposed model that Rga7 interacts with Rng10 through the BAR domain while with Ync13 through the GAP domain. We have added these clarifications to the revised manuscript (Results and Discussion) to better contextualize the evidence for the Rga7–Rng10–Ync13 assembly.

3. Liu Y, McDonald NA, Naegele SM, Gould KL, Wu J-Q. The F-BAR Domain of Rga7 Relies on a Cooperative Mechanism of Membrane Binding with a Partner Protein during Fission Yeast Cytokinesis. Cell Rep. 2019;26(10):2540-8.e4. doi: 10.1016/j.celrep.2019.01.112. PubMed PMID: 30840879; PubMed Central PMCID: PMCPMC6425953.
5. Liu Y, Lee I-J, Sun M, Lower CA, Runge KW, Ma J, et al. Roles of the novel coiled-coil protein Rng10 in septum formation during fission yeast cytokinesis. Mol Biol Cell. 2016;27(16):2528-41. Epub 2016/07/08. doi: 10.1091/mbc.E16-03-0156. PubMed PMID: 27385337; PubMed Central PMCID: PMCPMC4985255.

2) *It remains unclear whether Ync13 directly interacts with components of the glucan synthase complex (Bgs4/Ags1), or if this association is mediated through other factors (Rng10, Rga7). Clarifying the nature of this interaction would significantly enhance the mechanistic insight.*

Response: We thank the reviewer for this thoughtful clarification. As pointed out by Reviewer 1 in major comment 1, the multipass integral membrane proteins Bgs4 and Ags1 are embedded within vesicle membranes and are more likely to associate indirectly with the Rga7–Rng10-Ync13 complex rather than being part of one unified protein complex, although Rga7 Co-IPs with Bgs4 and its binding partner Smi1 (Figure 1, A-C). We would like to make it clear that our model or manuscript does not claim direct interactions between the Ync13-Rga7-Rng10 module and the glucan synthase complexes but suggest that the module aids in selection of vesicle targeting sites on the plasma membrane. To clarify, we have revised the text to more clearly state that our co-IP and in vitro binding results demonstrate that Rga7 physically associates with Ync13 and Rng10, and that vesicle-associated proteins such as Bgs4 and Ags1 are likely recruited through indirect interactions.

Minor comments:

1) *The manuscript refers to mass spectrometry-based interaction data, but the corresponding dataset is not included. Providing this would enhance transparency and reproducibility.*

Response: We apologize for the omission. The mass spectrometry data are now shown in Table S1.

2) *In Figure 2D, the MBP-6x pull-down lane shows a faint band around 76 kDa. The authors should clarify what this band represents and whether it has any relevance to the study.*

Response: We thank the reviewer for noticing this faint band. The weak ~76 kDa band in the MBP-6x pull-down lane is non-specific background binding of MBP and Rga7. We added a note in the figure legend to clarify this point.

3) *A quantification graph corresponding to the data in Figure 3G would aid in better interpreting the results and assessing their significance*.

Response: We thank the reviewer for this suggestion. We have now added two quantification graphs corresponding to Figure 3G, showing the measured Rng10 signal intensities across the division site. Statistical analysis shows the full width at half maximum (FWHM) is significantly different between WT and *ync13Δ* cells, and the figure legend and text have been updated accordingly in the revised manuscript.

4) *Figure 4D appears to be missing time legends, which are essential for interpreting the dynamics of the experiment*.

Response: We thank the reviewer for noticing this. We apology for making this confusing statement in figure legend. We would like to clarify that the full width at half maximum (FWHM) was calculated from line scans using single time point images from cells at the end of contractile-ring constriction. Those line scans were fitted with the Gaussian distribution to calculate the mean and standard deviation of FWHM. We have updated the figure legend to make it clearer in the revised manuscript.

Reviewer #2 (Significance (Required)):

Nature and Significance of the Advance
This study provides a conceptual and mechanistic advance in understanding the spatial and temporal regulation of membrane trafficking during cytokinesis. It identifies a conserved module-Ync13-Rga7-Rng10-that directs the selective tethering and fusion of secretory vesicles at the division site, functioning independently of the exocyst complex. This finding challenges the prevailing model that the exocyst is universally required for vesicle tethering during cytokinesis. While previous work has underscored the roles of TRAPP-II and vesicle trafficking in septum formation (Wang et al., 2016; Arellano et al., 1997; Gerien and Wu, 2018), the precise mechanism targeting vesicles to the division site remained unclear. This study fills that gap by elucidating how Ync13 and Rga7 coordinate vesicle delivery and glucan synthase localization (Liu et al., 2016; Zhu et al., 2018), thereby extending our understanding of septum biogenesis and membrane remodeling beyond actomyosin ring dynamics.

Relevant Audience:
This work is relevant to:
• Cell biologists investigating cytokinesis, membrane trafficking, or vesicle fusion.

• Yeast geneticists interested in conserved cell division pathways.
• Researchers focused on SNARE-mediated membrane dynamics and trafficking regulation.
• Biomedical scientists exploring analogous processes in mammalian systems, particularly those studying cell division defects linked to disease.

The findings have implications across both basic and translational research in cell biology and membrane dynamics.

My Expertise:

My research focuses on membrane fusion, specifically the SNARE-mediated fusion process. I study the spatio-temporal regulation of fusion events and the coordinated action of regulatory proteins in determining the structural and functional outcomes of membrane fusion. This background provides me with the framework to critically evaluate studies investigating cytokinesis and trafficking mechanisms at the molecular level.

Reviewer #3 (Evidence, reproducibility and clarity (Required)):

Zhang et al. elucidate key roles of a conserved module the Ync13-Rga7-Rng10 complex in coordinating selective tethering, docking, and fusion of glucan synthases containing vesicles with the plasma membrane, a process crucial for cell wall synthesis and survival of fission yeast at division. Using methods including mistargeting proteins to mitochondria, co-immunoprecipitation, in vitro binding assays, genetic and cellular methods, electron microscopy, and live-cell confocal microscopy, the authors demonstrate that this module controls a vesicle targeting pathway mediated by the TRAPP-II complex and SM protein Sec1, which ensures glucan synthases Bgs4 and Ags1 are deposited at the division site in a spatiotemporal manner.

Major comments:

The authors report aberrant accumulation of Bgs4 and Ags1 in the center of the septum after actomyosin ring constriction in ync13del cells and detect no overall defects in Bgs1 distribution there (Figure 4). When similar experiments were analyzed in this paper (https://pmc.ncbi.nlm.nih.gov/articles/PMC6249806/), Bgs1 distribution and level did change in cells lacking Ync13, although these phenotypes of Bgs1 appeared later that that of Bgs4. I wonder whether there could exist a second wave of Bgs1 arrival in ync13del cells at later time points after ring fully constricts. Could this late recruitment of Bgs1 depends on Rng7 and Rng10, since these protein complexes are enriched in the middle of septum of ync13del cells? Or as the authors mentioned in the Discussion, could Rho GTPase regulated by Rga7 GAP also play a role in Bgs1 accumulation or fusion with the septum in the above scenario, if no obvious accumulation of vesicles is observed in ync13del cells with electron microscopy? How does Bgs1 localize in ync13-19 rng10del double?

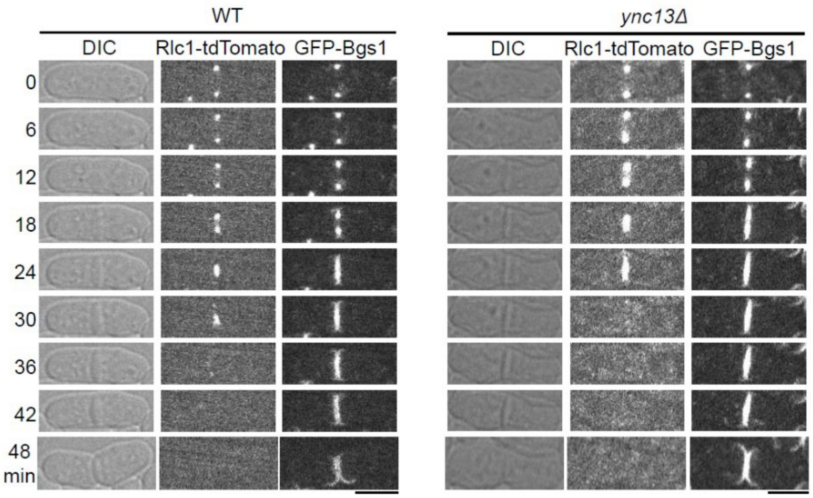

Response: We thank the reviewer for this insightful observation. We repeated the experiment to observe the localization of Bgs1 in WT and *ync13Δ* cells. We confirmed our earlier observation reported in this manuscript that the localization of Bgs1 at rim of the division site and its distribution along the division plane in *ync13Δ* is not very different from WT, although its intensity is higher and has more variation in *ync13Δ* cells (Figure above) . As suggested by the reviewer, we did microscopy to test Bgs1 localization in *ync13-19* temperature sensitive mutant, *rng10Δ*, *ync13-19 rng10Δ*, and WT (Fig. S7). While line scan curves for Bgs1 localization at the division site steep for *ync13-19 rng10Δ* double mutant, it has

no statistically significant difference in FWHM as compared to control WT (Fig. S7). Please note that we used different confocal systems, cameras, and laser powers for Fig. 4, C and E (PerkinElmer UltraVIEW Vox CSUX1) and Fig. S7 (Nikon W1+SoRa), so the FWHMs are not comparable between the two figures.

To test if there is any second wave of Bgs1 localization at the division site, we tracked the fluorescence intensity of Bgs1 throughout 2 h long movies and plotted the Bgs1 intensity profile at the division site over time. The data clearly show only one peak of Bgs1 and no later accumulation at the division site, although Bgs1 intensity has more variation in *ync13-19* and *ync13-19 rng10Δ* cells and the intensity is higher in *ync13-19 rng10Δ* cells. All these experiments conclude that Ync13-Rga7-Rng10 module impacts the localization of glucan synthases essential for the secondary septum (Bgs4 and Ags1) but not the primary (Bgs1).

Assessments of protein abundance by Western blotting (Figure 3C and 3D) can benefit from some quantifications.

Response: We thank the reviewer for this suggestion. We have now quantified the Western blot bands in Figures 3C and 3D, which have been added as supplementary figures along with the Western blot for Rng10 (Fig. S6, A-C) in the revised figures.

Minor comments:

Based on a series of experiments in which mistargeting Rga7 and Rng10 truncations drive Ync13-tdTomato to mitochondria, the authors suggest that Rga7, Rng10, and Ync13 have multivalent interactions with each other. Previous study (https://pmc.ncbi.nlm.nih.gov/articles/PMC6425953/) demonstrated that in cells co-expressing Tom20-GBP mECitrine-Rng10(751-950), Rga7 was efficiently mistargeted to mitochondria. This raises a possibility that Ync13 mistargeted by mECitrine-Rng10(751-1038) could come from Rga7 that strongly associated with Rng10(751-1038) on mitochondria. I wonder whether the authors could compare some of their truncation mistargeting experiments in the original manuscript and the ones in which either Rga7 or Rng10 is deleted, e.g. Tom20-GBP mECitrine-Rng10(751-1038) experiments in rga7del cells, if cells are still viable in this genetic background.

Response: We thank the reviewer for this insightful suggestion. We tested the mistargeting of mECitrine-Rng10(751–1038) in *rga7Δ tom20-GBP* cells and found that Ync13-tdTomato could not be recruited to mitochondria. This indicates that Ync13 cannot interact with Rng10 C-terminus independently of Rga7, supporting the Alphafold3 modeling and our proposed model that Rga7 interacts with Rng10 through the BAR domain while with Ync13 through the GAP domain. We have added the new data to the revised manuscript (Fig. S4H and associate text) and included a brief discussion highlighting that Rga7 is required for the Rng10–Ync13 interaction. We removed the mentioning of multivalent interactions in the manuscript to minimize confusion.

It is interesting that rga7del rng10del double mutants can survive better in EMM or YES with sorbitol. I wonder this would allow the authors to test whether the interaction between Ync13 and Sec1 is modulated by the presence of Rga7 and Rng10 or even the entire vesicle? Does mistargeted Ync13 overexpressed using the 3nmt1 promoter is still capable of driving Sec1 to mitochondria in rga7del rng10del cells.

Response: We thank the reviewer for this suggestion. While we did not succeed in constructing the pentamutant deleting both *rga7* and *rng10* and mislocalizing Ync13 to mitochondria, we were able to make a quadruple mutant deleting *rng10* and mislocalizing Ync13 to mitochondria. We tested whether mistargeted Ync13 overexpressed using the *3nmt1* promoter can recruit Sec1 to mitochondria in *rng10Δ* cells. Our results show that overexpressed Ync13 is still able to drive Sec1 localization to mitochondria without Rng10 (Fig. S2G). This suggests that Rng10 (together with Rga7) primarily functions to recruit and position Ync13 at the division site rather than being strictly required for the interaction between Ync13 and Sec1. This is also consistent with our Pmo25-GBP mislocalization experiments where we found that *rga7Δ 3nmt1-mECitrine-ync13* cells even under the repressed condition for the *3nmt1* promoter can partially rescue the lysis phenotype of *rga7Δ* cells (Figure 6).

The endogenous level of Ync13 is not particular high. Is this low level of Ync13 crucial for its function? Does mildly elevated level of Ync1 promote vesicle fusion at the closing septum?

Response: We thank the reviewer for this insightful question. To test if there is a correlation between Ync13 levels and vesicle fusion at the division site, we mildly overexpressed Ync13 from the *3nmt1* promoter in YE5S rich medium without additionally added thiamine to obtain cells with different Ync13 levels (the rich medium has some residual amount of thiamine, which partially represses the *nmt1* promoter) and then tracked the Rab11 GTPase Ypt3 labeled vesicles. This resulted in increased levels of Ync13 as well as Ypt3 at the division site (Fig. S8B). We measured the Ync13 intensity at division site and counted the number of Ypt3 vesicles reaching the division site in 2-minute continuous movie at the middle focal plane. We observed that increasing Ync13 level promoted the tethering and accumulation of Ypt3 vesicles at the division site until it reached a plateau (Fig. S8B). Thus, the Ync13 level is important for vesicle fusion at the division site. Collectively, Ync13, working with Rga7 and Rng10, plays an important role in vesicle targeting and fusion on the plasma membrane at the division site during cytokinesis. This is consistent with our results that overexpressed Ync13 can mislocalize Sec1 to mitochondria in *rng10Δ* (Fig. S2G) and can rescue the *rga7Δ* (Fig. 6).

Reviewer #3 (Significance (Required)):

Most of conclusions are well supported by a combination of methods. Out of curiosity, I wonder how much of Bgs4 or Smi1 detected in Co-IP experiments exist in the vesicle-bound form.
The authors propose a very interesting working model that addresses several key challenges in

achieving vesicle targeting specificity when timely delivery of various enzymes to their respective spatial locations along the primary and secondary septum must be orchestrated. I think this manuscript will be of interest to a broad audience.
